# SpiNeRF: direct-trained spiking neural networks for efficient neural radiance field rendering

**DOI:** 10.3389/fnins.2025.1593580

**Published:** 2025-07-23

**Authors:** Xingting Yao, Qinghao Hu, Fei Zhou, Tielong Liu, Zitao Mo, Zeyu Zhu, Zhengyang Zhuge, Jian Cheng

**Affiliations:** ^1^The Key Laboratory of Cognition and Decision Intelligence for Complex Systems, Institute of Automation, Chinese Academy of Sciences, Beijing, China; ^2^School of Future Technology, University of Chinese Academy of Sciences, Beijing, China; ^3^China Electric Power Research Institute Co., Ltd, Beijing, China

**Keywords:** spiking neural networks, neuromorphic computing, 3D rendering, neural radiance fields, efficient rendering, efficient SNN data encoding

## Abstract

Spiking neural networks (SNNs) have recently demonstrated significant progress across various computational tasks, due to their potential for energy efficiency. Neural radiance fields (NeRFs) excel at rendering high-quality 3D scenes but require substantial energy consumption, with limited exploration of energy-saving solutions from a neuromorphic approach. In this paper, we present SpiNeRF, a novel method that integrates the sequential processing capabilities of SNNs with the ray-casting mechanism of NeRFs, aiming to enhance compatibility and unlock new prospects for energy-efficient 3D scene synthesis. Unlike conventional SNN encoding schemes, our method considers the spatial continuity inherent in NeRF, achieving superior rendering quality. To further improve training and inference efficiency, we adopt a hybrid volumetric representation that allows the predefinition and masking of invalid sampled points along pixel-rendering rays. However, this masking introduces irregular temporal lengths, making it intractable for hardware processors, such as graphics processing units (GPUs), to conduct effective parallel training. To address this issue, we present two methods: Temporal padding (TP) and temporal condensing-and-padding (TCP). Experiments on multiple datasets demonstrate that our method outperforms previous SNN encoding schemes and artificial neural network (ANN) quantization methods in both rendering quality and energy efficiency. Compared to the full-precision ANN baseline, our method reduces energy consumption by up to 72.95% while maintaining comparable synthesis quality. Further verification using a neuromorphic hardware simulator shows that TCP-based SpiNeRF achieves additional energy efficiency gains over the ANN-based approaches by leveraging the advantages of neuromorphic computing. Codes are in https://github.com/Ikarosy/SpikingNeRF-of-CASIA.

## 1 Introduction

Spiking neural networks (SNNs) are considered as the third generation of neural networks (Maass, 1997; Roy et al., 2019). SNNs use neurons and synapses for computation. These components communicate via binary, asynchronous signals known as spikes. SNNs have attracted significant research interest over the last few years since their computing paradigm allows for theoretically sparse and low-power operations (Zhou et al., 2022; Zhu et al., 2022, 2023).

However, a significant gap remains between the anticipated potential of SNNs in advancing diverse efficient intelligence and the current dominance of artificial neural networks (ANNs) across most deep learning applications. A prominent example is high-quality 3D rendering, where ANNs have achieved impressive success but at the cost of substantial computational and energy overhead. For instance, NeRF (Mildenhall et al., 2021) achieves impressive rendering quality, yet demands massive energy consumption (Garbin et al., 2021). This inefficiency raises a critical question: *Can SNNs, with their event-driven and energy-efficient nature, enable high-quality 3D scene rendering while significantly reducing energy consumption?*

Li et al. (2025) were among the first to recognize the energy-saving potential of SNNs for efficient 3D rendering. However, their approach relies on the ANN2SNN conversion strategy (Diehl et al., 2016; Rueckauer et al., 2017), which results in an excessively large number of time-steps per sampled point, leading to significant energy redundancy. Therefore, their models fail to surpass or even match the energy efficiency of some optimized ANN works.^1^ In contrast, this paper explores NeRF rendering using directly trained SNNs and introduces a novel data encoding method designed to minimize time-steps, reduce energy consumption, and maintain high rendering quality.

Specifically, we adopt voxel grid methods (Hedman et al., 2021; Liu et al., 2022; Sun et al., 2022), which explicitly store volumetric parameters and allow predefined masking of invalid sampled points, to enable fast and efficient synthesis. For more energy-efficient computation, we use a directly trained spiking multilayer perceptron (MLP) that implicitly encodes volumetric parameters through a spike-driven approach with a minimal number of time-steps. By combining these explicit and implicit strategies, our SNN-based NeRF (named SpiNeRF) achieves both fast and energy-efficient neural radiance rendering.

Inspired by the imaging process of the primate fovea where photoreceptor cells are stimulated by the accumulation of light intensity over time (Masland, 2012; Wässle, 2004) we draw a comparison between the accumulation process in NeRF rendering and the temporal integration in SNNs that stimulates spiking activity. Concretely, we align each pixel-rendering ray with the temporal dimension of the spiking MLP, mapping each sampled point along the ray to a corresponding time-step during rendering. This approach transforms the geometric consecutiveness of the ray into temporal continuity within the SNN. Thus, SpiNeRF can achieve higher rendering quality and better energy efficiency when compared to previous data encoding methods.

Moreover, since invalid sampled points are masked out, the number of sampled points along different pixel-rendering rays varies, resulting in irregular temporal lengths across different rays. Consequently, querying volumetric parameters becomes challenging to parallelize during rendering, which significantly hinders the training process on graphics processing units (GPUs). To solve this issue, we first investigate the temporal padding (TP) method, which standardizes the temporal length within a querying batch (i.e., creates a regular-shaped tensor), ensuring parallelism and enabling efficient GPU-based training. Furthermore, we propose a temporal condensing-and-padding (TCP) strategy, which further reduces tensor size and condenses the data distribution, making the approach more hardware-friendly for both neuromorphic hardware and GPUs. Extensive experiments prove that TCP effectively maintains the energy efficiency advantages of SNNs while maintaining high-quality NeRF rendering.

Additionally, we discuss the querying direction of SpikNeRF since SNNs process temporal information in a particular direction while the accumulation process of the NeRF rendering does not.

In summary, the main contributions of this work are as follows:

- We propose SpiNeRF, a novel framework that aligns the temporal dimension of SNNs with the pixel-rendering rays of NeRF, effectively leveraging the temporal characteristics of SNNs. To the best of our knowledge, this is the first work to employ directly trained SNNs for reconstructing 3D RGB scenes, enabling efficient and high-quality 3D rendering feasible on neuromorphic hardware.
- We introduce TP and TCP to solve the issue of irregular temporal lengths, enabling parallelism during training and inference on GPUs. In particular, TCP further enhances hardware compatibility, making SpiNeRF efficient on both neuromorphic hardware and GPUs.
- We validate the effectiveness of SpiNeRF across four mainstream tasks, demonstrating significant energy efficiency advancements. For example, on the Tanks&Temples task, SpiNeRF achieves a 72.95% reduction in energy consumption with only a 0.33 dB drop in PSNR.

This paper is structured as follows: Section 2 reviews related work, Section 3 outlines the preliminaries, Section 4 details the proposed methods, Section 5 presents the results, and Section 6 concludes the study.

## 2 Related work

### 2.1 NeRF-based 3D rendering

In contrast to traditional 3D rendering methods that rely on explicit and discrete volumetric representations, NeRF (Mildenhall et al., 2021) utilizes a coordinate-based neural network to implicitly represent the 3D radiance field. It synthesizes novel views by accumulating density and color information along view-dependent rays using a ray tracing algorithm (Kajiya and Von Herzen, 1984). This paradigm has significantly improved the quality of novel view synthesis. Subsequent works have enhanced rendering quality (Deng B. et al., 2020; Barron et al., 2021; Tancik et al., 2020), while others have focused on accelerating training (Deng et al., 2022; Fridovich-Keil et al., 2022; Sun et al., 2022) or optimizing the rendering process itself (Lindell et al., 2021; Reiser et al., 2021; Yu et al., 2021; Sun et al., 2022). In this work, we explore the integration of spike-based, low-energy computation with NeRF-based high-quality 3D synthesis, aiming to enable energy-efficient and neuromorphic-hardware-compatible 3D rendering.

### 2.2 Fast NeRF synthesis

The accumulation process in NeRF (Mildenhall et al., 2021) involves a large number of MLP queries, resulting in substantial flop operation and memory-access overhead, which slows down the synthesis speed. To address this, recent studies have introduced hybrid models that combine traditional explicit volumetric representations such as voxels (Hedman et al., 2021; Liu et al., 2022; Sun et al., 2022) and MPIs (Wizadwongsa et al., 2021) with MLP-dependent implicit representations, thereby improving efficiency by reducing redundant queries in free space. In this work, we adopt voxel grids to mask out irrelevant low-density regions and discard unimportant sampled points with low weights, significantly reducing synthesis overhead. Notably, our proposed method based on a novel adaptation of SNNs is designed as a plug-in component that remains orthogonal to existing ANN-based NeRF models.

### 2.3 Spiking neural networks

Due to their high sparsity and multiplication-free operations, SNNs outperform ANNs in potential energy efficiency (Davies et al., 2018; Li et al., 2020; Lee et al., 2022). However, they have historically lagged behind in performance. To bridge this gap, research has focused on deepening SNN architectures (Zheng et al., 2021; Fang et al., 2021a), accelerating convergence (Wu et al., 2019; Deng et al., 2021), and achieving high performance (Zhou et al., 2022). With advancements in both energy efficiency and model performance, recent research has explored more versatile SNN models, including Spikformer (Zhou et al., 2022), Spiking GCN (Zhu et al., 2022), and SpikeGPT (Zhu et al., 2023). In this paper, we seize the analogous nature of NeRF and SNNs to enable spiking neural networks to reconstruct 3D scenes with high quality at low energy consumption.

### 2.4 SNNs in 3D reconstruction

The application of SNNs to 3D reconstruction remains relatively unexplored. Spiking-NeRF (Li et al., 2025) represents one of the first attempts to achieve efficient NeRF rendering using SNNs as the fundamental model. However, their approach relies on the ANN2SNN conversion strategy (Diehl et al., 2016; Rueckauer et al., 2017), which leads to excessively long temporal lengths for each sampled point, resulting in superfluous energy consumption. In contrast, our method directly trains SNN models from scratch, reducing the number of time-steps per sampled point to 1. Consequently, our approach achieves orders of magnitude lower energy consumption compared to previous works.

Additionally, Liao et al. (2023) propose a non-linear, non-spiking function, B-FIF, to post-process the density estimation of the original ANN-based NeRF (Mildenhall et al., 2021). While this method may enhance depth prediction, it abandons the binary spiking nature and potential energy efficiency of SNNs contradicting the core motivation of our work. Moreover, their evaluation is limited to the Chamfer metric, which does not support comprehensive quantitative or qualitative comparisons with our method, which targets full RGB scene rendering.

## 3 Preliminaries

### 3.1 Neural radiance field

To reconstruct view-dependent colors of a given scene, NeRF (Mildenhall et al., 2021) utilizes an MLP that takes in the location coordinates **p**∈ℝ^3^ and the view direction **v**∈ℝ^2^, and outputs the density σ∈ℝ and the color **c**∈ℝ^3^. This MLP implicitly maintains a continuous volumetric representation:


(1) $$\begin{array}{ll} \text{e},\sigma & =MLP_{\theta }\left(\text{p}\right), \end{array}$$

(2) $$\begin{array}{ll} \text{c} & =MLP_{\gamma }\left(\text{e},\text{v}\right), \end{array}$$

where θ and γ denote the parameters of the two separate parts of the MLP, and **e** denotes the embedded features. Next, NeRF renders each pixel of the desired view by casting a pixel-rendering ray **r** from the camera origin toward the corresponding pixel direction and sampling *K* points along this ray. Through querying the MLP *K* times, as described in Equations 1, 2, *K* color values and *K* density values are obtained. Finally, following the principles of discrete volume rendering proposed by Max (1995), the expected RGB color of the pixel, Ĉ(**r**), is computed as follows:


(3) $$\alpha_{i}=1-\exp(-\sigma_{i}\delta_{i}),\;w_{i}=\prod_{j=1}^{i-1}\left(1-\alpha_{i}\right),$$

(4) $$\hat{C}(r)\approx \sum_{i=1}^{K}w_{i}\alpha_{i}c_{i},$$

where **c**_*i*_ and σ_*i*_ denote the color and density values of the *i*-th sampled point, respectively; δ_*i*_ is the distance between adjacent points *i* and *i*+1, and α_*i*_ is an intermediate variable used in volume rendering.

After rendering all the pixels, the expected view is reconstructed. Given the ground-truth pixel color *C*(**r**), the MLP parameters can be trained end-to-end by minimizing the MSE loss:


(5) $$ℒ=\frac{1}{\left|ℛ\right|}\sum_{r\in ℛ}\|\hat{C}(r)-C(r){\|}_{2}^{2},$$

where R is the mini-batch containing the sampled rays.

### 3.2 Hybrid volumetric representation

The number of sampled points *K* in Equation 4 is usually large, leading to a heavy MLP querying burden, as displayed in Equations 1, 2. To alleviate this problem, voxel grid representations (Liu et al., 2020; Sun et al., 2022) are utilized to directly store volumetric parameters such as the embedded feature *e* and density σ from Equation 1 as values within the grid. Therefore, the costly MLP queries in Equation 1 are replaced with simpler voxel grids and interpolation operations, significantly reducing computational overhead:


(6) $$\sigma =\text{act}(\text{interp}(p,V_{\sigma })),$$

(7) $$e=interp(p,V_{f}),$$

where **V_σ_** and **V_*f*_** denote the voxel grids corresponding to volumetric density and features, respectively. The operator “interp” denotes the interpolation operation, while “act” represents an activation function, such as ReLU or the shifted softplus (Barron et al., 2021).

Furthermore, irrelevant points with low density or unimportant points with low accumulated weight can be masked using predefined thresholds λ. Therefore, Equation 4 is modified to:


(8) $$\begin{array}{l} A≜\left\{i:w_{i}>\lambda_{1},\alpha_{i}>\lambda_{2}\right\}, \end{array}$$

(9) $$\hat{C}(r)\approx \sum_{i\in A}w_{i}\alpha_{i}c_{i}.$$

Thus, the number of MLP queries for sampled points, as shown in Equation 2, is significantly reduced. These computational benefits make hybrid volumetric representation widely used in neural radiance rendering (Sun et al., 2022; Chen et al., 2022).

### 3.3 Spiking neuron

The spiking neuron is the most fundamental unit of SNNs and serves as the key distinguishing factor between SNNs and ANNs. Spiking neurons are commonly adopted using the leaky integrate-and-fire (LIF) model:


(10) $$\begin{array}{ll} u_{j}^{t} & =\left(1-\frac{1}{\tau }\right)v_{j}^{t-1}+\frac{1}{\tau }\underset{i}{\sum }w_{ij}s_{i}^{t}+\frac{1}{\tau }v_{reset}, \end{array}$$

(11) $$\begin{array}{ll} s_{j}^{t} & =ℍ\left(u_{j}^{t}-v_{thr}\right), \end{array}$$

(12) $$\begin{array}{ll} v_{j}^{t} & =u_{j}^{t}\cdot \left(1-s_{j}^{t}\right)+v_{reset}s_{j}^{t}. \end{array}$$

In this work, we adopt the LIF neuron implementation provided by the renowned SpikingJelly framework (Fang et al., 2023a). The membrane potential of neuron *j* at time-step *t*, denoted as $u_{j}^{t}$, is updated according to Equation 10, where $v_{j}^{t-1}$ represents the post-spike membrane potential, and *w*_*ij*_ is the synaptic weight from neuron *i* to neuron *j*. The output spike $s_{j}^{t}$ is determined by the Heaviside step function ℍ(·) in Equation 11, which triggers a spike when the membrane potential exceeds the potential threshold *v*_*thr*_. Depending on whether a spike is emitted at time-step *t*, the post-spike membrane potential $v_{j}^{t}$ is either retained as $u_{j}^{t}$ or reset to a fixed value *v*_*reset*_, as described in Equation 12.

Since the Heaviside step function ℍ(·) is not differentiable, we use the surrogate gradient method (Neftci et al., 2019) to solve this issue:

In the forward pass,


(13) $$ℍ(x)=\{\begin{array}{l} 1,x\geq 0,\\ 0,\text{otherwise.} \end{array}$$

In the backward pass,


(14) $$\begin{array}{l} \frac{dℍ\left(x\right)}{dx}\approx \frac{d\text{ Sigmoid}\left(\beta x\right)}{dx}=\frac{\beta \exp\left(-\beta x\right)}{{\left(1+\exp\left(-\beta x\right)\right)}^{2}}, \end{array}$$

where β is a predefined hyper-parameter. This allows spiking neural networks to be optimized in an end-to-end manner.

## 4 Methods

This section describes the process of constructing an efficient NeRF using directly trained SNNs. First, we explore two conventional SNN encodings (direct encoding and Poisson encoding) to adapt SNNs for NeRF. We also consider ANN quantization as an encoding strategy for building efficient NeRF models. These encodings will serve as comparative baselines. Next, on the basis of direct encoding, we propose the time-ray alignment encoding, which integrates the sequential processing capabilities of SNNs with the ray-casting mechanism of NeRF. Following this, we develop TP to handle the irregularities introduced by the masking operation along the temporal dimension. We further propose TCP to create a denser data structure, making it more compatible with hardware computation. We also address the alignment direction issue and propose the temporal flip trick as part of our empirical study. Lastly, we provide the network architecture, the overall algorithm, and the experimental setups to finish the practical establishment of SpiNeRF and ensure the reproducibility of our results.

### 4.1 Data encoding

**Figure 1:**
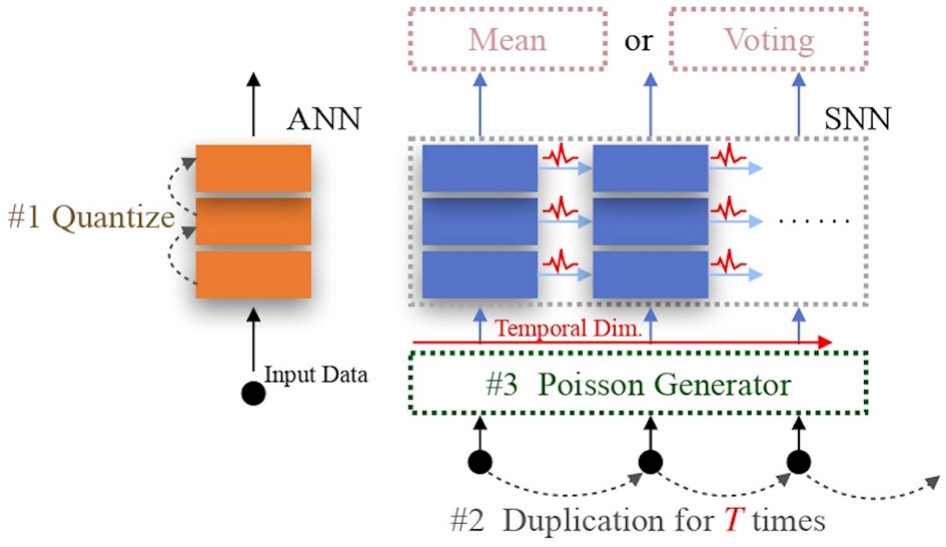
Conventional encoding schemes. For ANN quantization #1, 16-bit or 32-bit float point features are mapped to low-precision values. For direct encoding, only the operation #2 is necessary that it duplicates the input data *T* times to fit the length of the temporal dimension. For Poisson encoding, both operation #2 and #3 are utilized to generate the input spike train. The “Mean” or “Voting” operation is able to decode the SNN output.

In this subsection, we explore one classic ANN data/feature quantization method and two conventional SNN data encoding approaches: direct encoding and Poisson encoding, as initial strategies for efficient NeRF rendering. ANN quantization encodes data/features in a low-precision format, reducing hardware computing overhead during MAC operations. The two conventional SNN encodings are designed to facilitate the practical application of SNNs, reducing computing overhead. Therefore, these three data encoding strategies represent viable pathways to achieving efficient NeRF rendering. It is logical to first establish these baseline models as a foundation for further exploration. Figure 1 illustrates their operating processes.

For ANN quantization, we adopt the renowned Learned Step-size Quantization (LSQ) (Esser et al., 2020) to quantize the original ANN model, thereby establishing an efficient ANN-based NeRF model as the ANN encoding.

In the direct encoding scheme, the original data is duplicated *T* times to match the length of the SNN's temporal dimension, where *T* represents the total length of the temporal dimension. In the Poisson-encoding scheme, besides the duplication operation, the input value is interpreted as the probability of generating a spike at each time-step. A decoding method is also required for the subsequent rendering operations, with the mean (Li et al., 2021) and voting (Fang et al., 2021b) decoding operations being commonly considered. We employ the former approach, as the voting method is designed for classification tasks (Diehl and Cook, 2015; Wu et al., 2019). Thus, we create two baseline versions of SpiNeRF using these two SNN encoding methods.

### 4.2 Time-ray alignment encoding

This subsection explores a more natural and novel approach to adapting SNNs for the NeRF rendering process. Specifically, attempt to retain the real-valued input data, as in direct encoding, but avoid using the duplication-based approach to fill the temporal dimension.

We first consider the MLP querying process from the ANN philosophy. To reconstruct the expected view, the volumetric parameters of the sampled points, such as **e** and **v** in Equation 2, are organized as input data with shapes of [*batch, c*_*e*_] or [*batch, c*_*v*_], where *batch* represents the sample index and *c* is the channel index of the volumetric parameters. This structure allows the MLP to query these data and output the corresponding color information in parallel. However, from a geometric view, the input data should be structured as [*ray, pts, c*], where *ray* represents the ray index and *pts* represents the index of the sampled points.

Clearly, the ANN-based MLP querying process does not capture the geometric relationships between the pixel-rendering ray and the sampled points. To address this, we consider the computational modality of SNNs. As illustrated in Equations 10–12, SNNs involve the temporal dimension to process sequential signals. This means that a spiking MLP naturally accepts input data with the shape of [*batch, time, c*], where *time* refers to the temporal index. Therefore, we can reshape the volumetric parameters back to [*ray, pts, c*], and intuitively match each sample along the ray to the corresponding time-step:


(15) $$\begin{array}{l} {\text{Input}}_{MLP}:=[batch,c]\\ \;\Rightarrow [ray,pts,c]\\ \;\Rightarrow [batch^{\prime },time,c]:={\text{Input}}_{Spiking_{\_}MLP}. \end{array}$$

**Figure 2:**
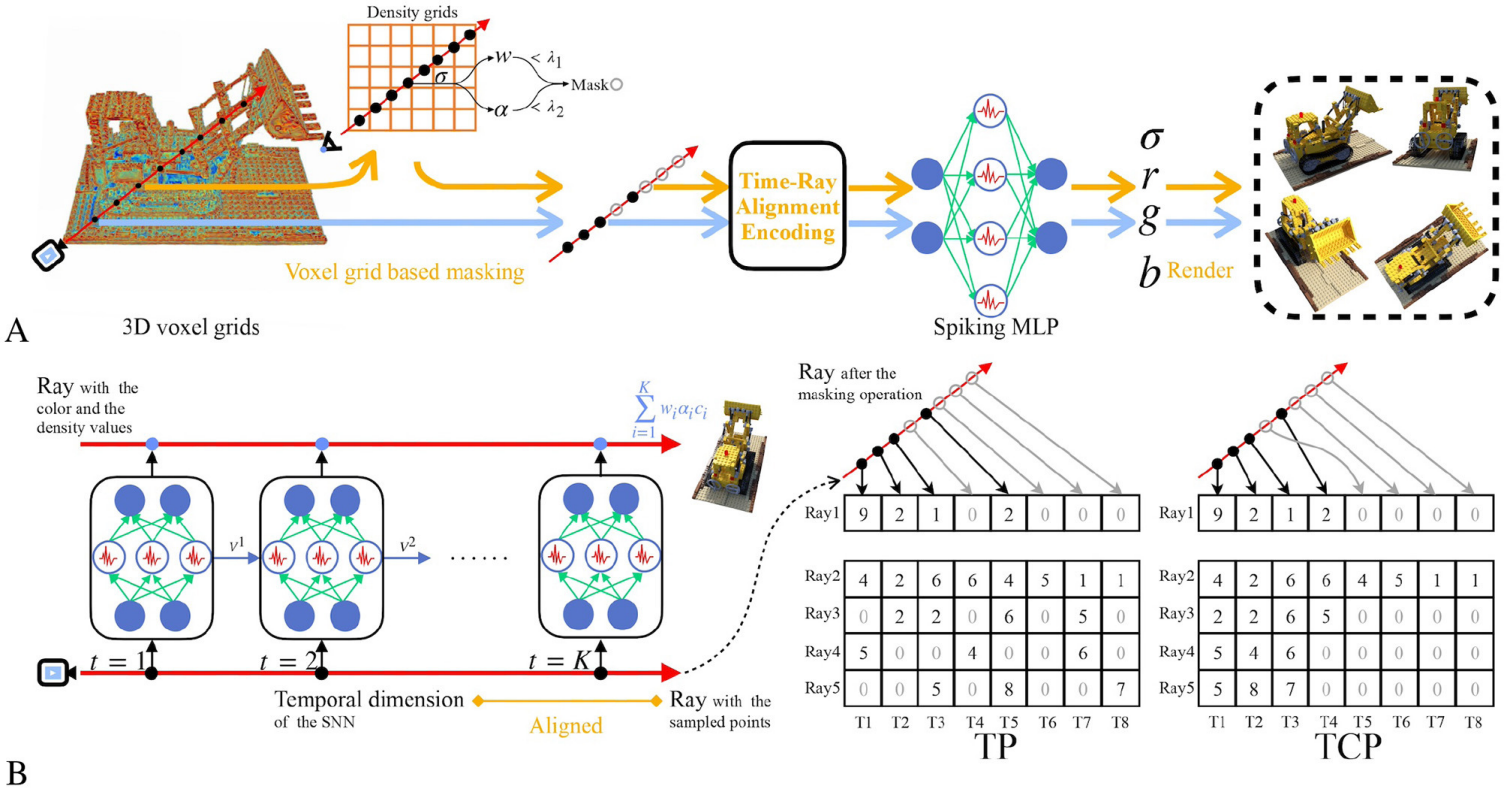
The overview of the proposed SpiNeRF. **(A)** The rendering process of SpiNeRF. The whole 3D volumetric parameters are stored in the voxel grids. The irrelevant or unimportant samples are masked before the spiking MLP querying. The expected views are rendered with the volumetric information yielded by the spiking MLP. **(B)** The proposed time-ray alignment encoding, where the temporal dimension and the pixel-rendering ray are aligned. The spiking MLP queries each sampled point step-by-step to yield the volumetric information. The proposed temporal padding (TP) method or the temporal condensing-and-padding (TCP) method can be performed before the spiking MLP querying. For simplification, the channel length of the volumetric parameters is set to 1.

Thus, *ray* = *batch*′ and *pts* = *time*, which indicates the geometric relationships of sampled points can be captured by SNNs in the form of temporal intervals. This is further illustrated in Figure 2A. Notably, this alignment does not require any input data pre-process, such as duplication (Zhou et al., 2022) or Poisson generation (Garg et al., 2021), which are commonly used in previous works.

### 4.3 TCP

However, the masking operation on sampled points, as illustrated in Section 3.2, complicates the time-ray alignment. While this masking operation improves rendering speed and quality by curtailing the computation cost of redundant samples, it also leads to an irregular number of queried samples across different rays. Therefore, the reshape operation in Equation 15, which transforms the data into a tensor, becomes unfeasible on GPUs after the masking operation.

To ensure computation parallelism (particularly during training) on GPUs, we propose retaining the indices of masked samples while discarding their values. As illustrated in Figure 2B, both unmasked and masked samples are sequentially arranged in the corresponding *ray*-indexed vector, with zeros padded into the vacant tensor elements. This results in a regular-shaped input tensor. We refer to this simple approach as the temporal padding (TP) method.

However, the TP method does not effectively handle masked samples because the padded zeros still participate in subsequent computation, causing the membrane potential of the spiking MLP to decay. This implicitly affects the outcomes of the unmasked samples in the posterior segment of the ray. What's worse, these zeros interspersed among valid data elements increase the irregular local sparsity of the tensor. Even with advanced hardware architectures that can skip these zeros, the sparse data structure still causes computation inefficiency, such as imbalanced workload (Zhang et al., 2020). To solve these issues, we propose the temporal condensing-and-padding (TCP) strategy, as illustrated in Figure 2B. In contrast to TP, the TCP strategy completely discards the parameters and indices of the masked samples, rearranging the unmasked sampled points adjacently in the corresponding *ray* vector. Thus, the data density can be locally increased. Additionally, the *ray* dimension can be sorted according to the number of valid data points, further increasing the valid data density. For example, if the *ray* dimension is sorted in descending order of valid data, the valid data will be concentrated in the upper-left part of the tensor. This sorting trick is also incorporated into our TCP technique to facilitate more efficient allocation of computation resources, avoiding redundant computation on invalid data. Therefore, TCP effectively eliminates the impact of masked samples, making SpiNeRF more hardware-friendly.

Although the data condensing operation may introduce additional overhead on hardware, the resulting regular and condensed data structure commonly yields significantly greater efficiency gains, outweighing the extra cost of data condensing (Zhang et al., 2020). These benefits extend beyond DNN hardware accelerators and GPUs to neuromorphic hardware as well, as demonstrated in Parallel Time Batching (PTB) (Lee et al., 2022) and STELLAR (Mao et al., 2024). Therefore, we adopt TCP as the primary method in the following study.

### 4.4 Temporal flip

Moreover, aligning the temporal dimension with the pixel-rendering ray causes the spiking MLP to query the samples along each ray sequentially rather than in parallel. This raises an important question: Which querying direction is more efficient for SpiNeRF, the original pixel-rendering ray direction or its reverse?

**Figure 3:**
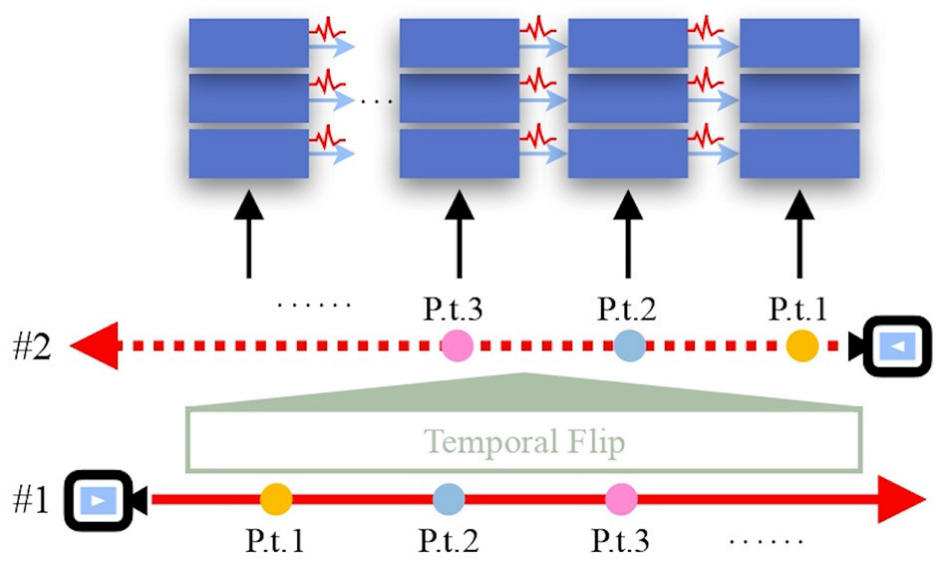
Temporal flip. The direction of the temporal dimension is consistent with the pixel-rendering ray #1 but opposite to ray #2. “P.t.” is the abbreviation of “point”.

Although some special SNN designs, such as Parallel Spiking Neuron (PSN) (Fang et al., 2023b), can process the parallel input data and avoid the querying direction issue, our goal is to find a general solution that adapts standard, one-directional SNNs to SpiNeRF. To address this, we propose using empirical experiments to determine the optimal querying direction. As illustrated in Figure 3, we introduce a temporal flip operation that reverses the alignment between the temporal dimension and the pixel-rendering ray, effectively enabling a direct comparison between the two directions. Experimental results presented in Section 5.4 consistently indicate that the direction of the pixel-rendering ray yields better performance for SpiNeRF. Therefore, we adopt this direction as the default in our method.

### 4.5 Network architecture

As illustrated in the above subsections and Figure 2, the proposed SpiNeRF integrates three-folded techniques: time-ray alignment (TRA) encoding, TCP, and the replacement of ANN with SNN. Together, these techniques enable SpiNeRF to operate as a plug-in module, improving the potential energy efficiency of voxel-grid-based NeRF frameworks. As an example, we incorporate our method into the Direct Voxel Grid Optimization (DVGO) framework (Sun et al., 2022), resulting in a variant termed SpiNeRF-D. This subsection introduces an optional spiking MLP architecture, which serves as the main method used in our experiments.

The spiking MLP comprises an input projection layer (39 → 128 channels), a hidden layer (128 → 128 channels), and an output layer (128 → 3 channels). All layers are implemented as bias-free linear transformations followed by LIF spiking neurons. The network architecture setting and parameter size remain identical to those in the original ANN-based model (Sun et al., 2022), with the sole modification being the replacement of ReLU functions with LIF spiking neurons. As indicated in the architecture, the output feature has 3 channels corresponding to the RGB values of **c** in Equation 2. After applying Equation 9, the final RGB values for each pixel Ĉ(**r**) are produced. The detailed rendering pipeline is described in the following subsection.

### 4.6 Overall algorithm

This subsection summarizes the overall SpiNeRF algorithm within the DVGO framework (Sun et al., 2022), referred to as SpiNeRF-D. The pseudo code for inference, i.e., the rendering process, is provided in Algorithm 1, while the training pipeline is described in Algorithm 2.

**Algorithm 1:**
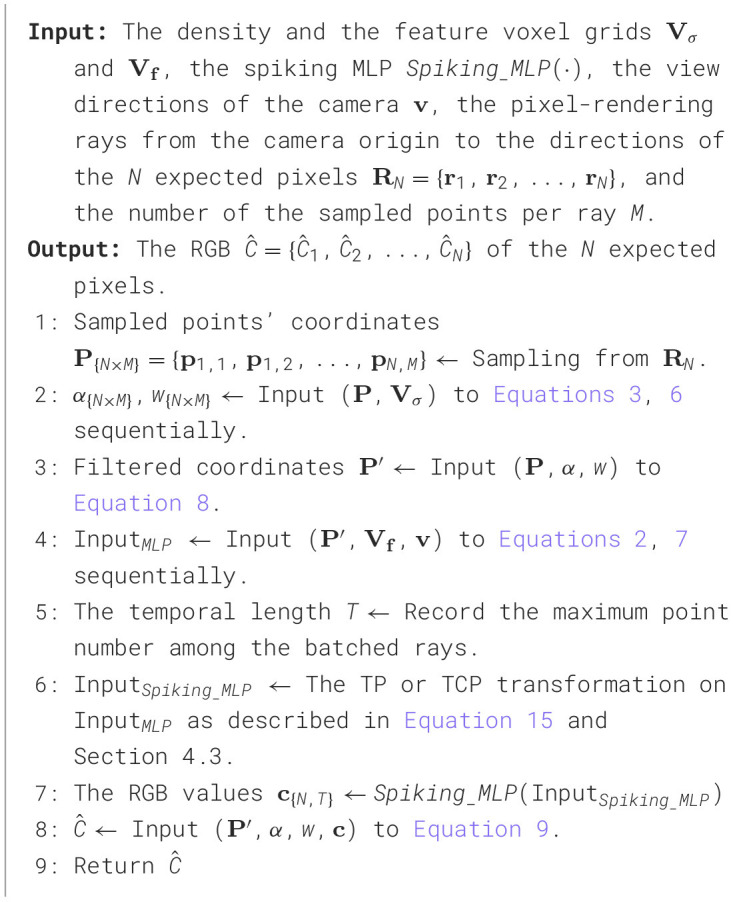
Rendering pipeline of SpiNeRF-D.

**Algorithm 2:**
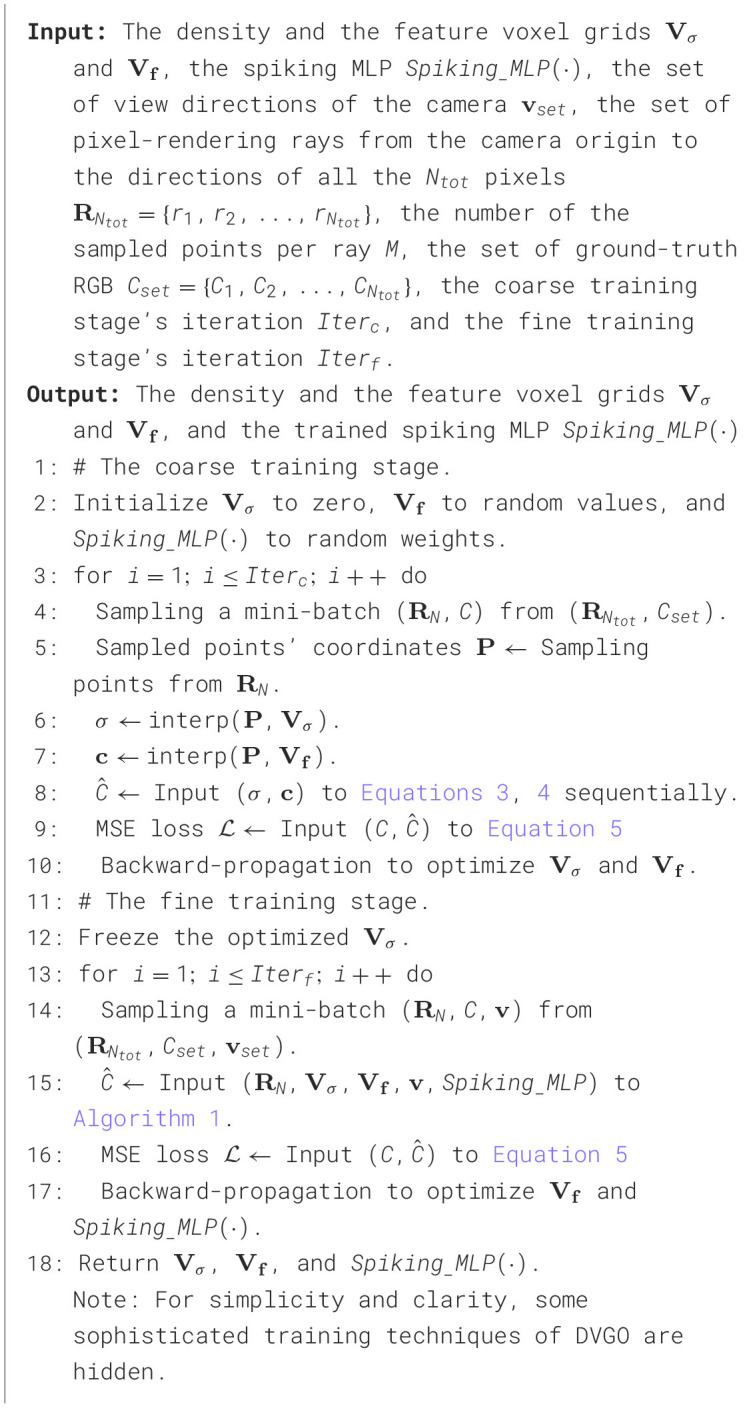
Training pipeline of SpiNeRF-D.

As illustrated in Figure 2A, SpiNeRF-D first establishes the voxel grids filled with learnable volumetric parameters. Two groups of voxel grids are built as the input of Algorithm 1, which are the density and the feature voxel grids. Given the *N* expected pixels to render, Step 1 is to sample *M* points along each pixel-rendering ray shot from the camera origin to the direction of each pixel. With the *N*×*M* sampled points, Step 2 queries the density grids to compute the weight coefficients, and Step 3 uses these coefficients to mask out those irrelevant points. Then, Step 4 queries the feature grids for the filtered points and returns each point's volumetric parameters. Steps 5 and 6 prepare the volumetric parameters into a receivable data format for *Spiking*___*MLP* with TP or TCP. Steps 7 and 8 compute the RGB values for the *N* pixels. Step 9 returns these *N* rendered pixels. Assuming these *N* pixels all belong to a $\sqrt{N}\times \sqrt{N}$ sized novel view **v**, this novel view can then be rendered by putting together all its *N* pixels.

In the training pipeline of SpiNeRF-D, we adopt the two-stage training strategy used in the original DVGO framework: a coarse training stage followed by a fine training stage. The purpose of the coarse training stage is to provide better initialization for the two voxel grids **V_σ_** and **V_*f*_**. As shown in Algorithm 2, only these two voxel grids are optimized during the coarse training stage. In Steps 6 and 7, the density σ and color **c** are directly extracted from the voxel grids. Step 8 generates the coarse RGB values for the expected pixels, which are then compared with ground truth in Step 9 to compute the loss. Finally, Step 10 performs a backward pass to optimize **V_σ_** and **V_*f*_**.

After optimizing **V_σ_** and **V_*f*_** for *Iter*_*c*_ iterations during the coarse training stage, the fine training stage will leverage these properly initialized voxel grids to accelerate convergence. In this stage, Step 12 freezes the density voxel grid **V_σ_**, treating it as a fixed parameter. Step 15 leverages the spiking MLP for refined pixel rendering, following Algorithm 1. Using the predicted pixel values and ground truth, the MSE loss is calculated in Step 16, enabling a backward pass in Step 17. After *Iter*_*f*_ iterations of fine training, SpiNeRF-D achieves high-quality 3D rendering with an optimized spiking MLP and an enhanced **V_*f*_**. Moreover, the fine training stage accounts for over 95% of the total training time due to its high iterations and the additional operations introduced by the spiking MLP. This substantiates the rationale for employing the proposed TCP module to maintain training efficiency.

Notably, the proposed methods, which function as a plug-in, can also be applied to other voxel-grid-based NeRF frameworks, such as TensoRF (Chen et al., 2022) and NSVF (Liu et al., 2020). Therefore, we also implement SpiNeRF within the TensoRF framework (termed SpiNeRF-T) to further verify its generalizability in Section 5.

### 4.7 Method verification setup

#### 4.7.1 Dataset benchmarks

We conduct experiments on four inward-facing datasets: Synthetic-NeRF (Mildenhall et al., 2021), which contains eight objects synthesized from realistic images; Synthetic-NSVF (Liu et al., 2020), containing eight objects generated by NSVF; BlendedMVS (Yao et al., 2020), which includes authentic ambient lighting by blending real images; and the real-world dataset Tanks&Temples (Knapitsch et al., 2017).

#### 4.7.2 Hyper-parameter setting

In SpiNeRF-D, we retain all the hyper-parameters from the original configuration (Wu et al., 2019), except for increasing the number of fine training iterations *Iter*_*f*_, from 20,000 to 40,000. This adjustment helps address the underfitting issue commonly observed in SNNs, which requires more training iterations to resolve (Fang et al., 2021a,b). The comparisons between SpiNeRF-D and DVGO are fair, as we also set the fine training iteration count to 40,000 for our reproduced DVGO. All other hyper-parameters remain unchanged, including 5,000 iterations for the coarse stage training *Iter*_*c*_, 8192 rays per training batch, and a 160^3^ voxel grid size. For both SpiNeRF-T and our reproduced TensorRF, we discard the feature embedding to alleviate the first layer's computation burden. Aside from this modification, all other hyper-parameters follow the official TensorRF configurations (Chen et al., 2022).

#### 4.7.3 Energy estimation

For energy computation, we adopt two estimation methods. The first method is intended for comparison with previous works and to align our results with the major SNN field. Specifically, we follow previous works (Zhou et al., 2022; Yao et al., 2023; Kundu et al., 2021a,b; Horowitz, 2014) by providing a theoretical energy consumption estimate based on 45 nm technology (Horowitz, 2014) and report the average energy consumption required to render a novel view. The energy cost of spike-based operations is defined as follows: Energy_SOPs_ = 0.9*pJ*×*Spike*_*num*×*Flops*, where *Spike*_*num* denotes the number of spikes in the input spike train, and *Flops* represents the number of floating-point operations triggered by a single spike. Similarly, the energy consumption for the floating-point operations is estimated as follows: Energy_FLOPs_ = 4.6*pJ*×*FLOPs*. The total energy consumption for rendering a novel view, denoted as Energy_tot_, is calculated as: Energy_tot_ = *Pts*_*num*×(Energy_SOPs_+Energy_FLOPs_), where *Pts*_*num* denotes the total number of sampled points. Unless specified otherwise, this energy estimation method is used by default.

The second energy estimation method estimates the effectiveness of our proposed approach on neuromorphic hardware architecture. Specifically, we adopt the SpikeSim evaluation with the SpikeFlow architecture (Moitra et al., 2023). SpikeSim emulates neuromorphic hardware design based on in-memory-processing technology, while SpikeFlow defines the default from-software-to-hardware network mapping and dataflow configuration. In SpikeSim, model parameters are stored as conductance values in crossbars within each processing unit. Each crossbar performs a matrix-vector multiplication, and each processing unit handles multiple such operations in parallel. Since the spiking MLP in SpiNeRF-Ds is relatively small, we reduce the crossbar size from the original 64 × 64 to 32 × 32, making the mapping of the spiking MLP to the SpikeFlow architecture feasible. Additionally, the original SpikeFlow architecture was designed for image classification tasks and does not consider dataflow parallelism, as the original model size is large. In our implementation, we set the parallelism of dataflow to 4, meaning that four spiking MLPs are stored on-chip and four pixel-rendering rays in the radiance field can be queried simultaneously by these spiking MLPs. This setup enables feasible energy evaluation using SpikeSim.

## 5 Results

In this section, we demonstrate the effectiveness of our proposed SpiNeRF. First, we base our implementation on the voxel-grid-based DVGO (Sun et al., 2022) as the basic NeRF framework for our experiments. Second, we then compare the proposed TRA encoding with existing data encoding methods, including ANN quantization (Esser et al., 2020), Poisson encoding, and direct encoding, to demonstrate the effectiveness and superiority of TRA. Third, we compare our methods with other NeRF-based methods, including traditional ANN counterparts and the ANN2SNN-based Spiking-NeRF (Li et al., 2025). To demonstrate the flexibility of our approach, we also integrate our methods into the high-performance TensoRF framework (Chen et al., 2022) and showcase the corresponding results. In addition, we present rendering views as visual aids for qualitative analysis. Fourth, the neuromorphic hardware simulator SpikeSim (Moitra et al., 2023) and an A100 GPU are used to verify the efficiency of the proposed TCP strategy. The results collectively demonstrate the hardware compatibility of the proposed methods. Finally, we discuss the impact of the alignment direction by applying the temporal flip technique, as described in Section 4.4.

For clarity, we refer to the DVGO-based implementation of SpiNeRF as SpiNeRF-D, and the TensoRF-based implementation as SpiNeRF-T.

### 5.1 Comparisons with the conventional data encodings

**Figure 4:**
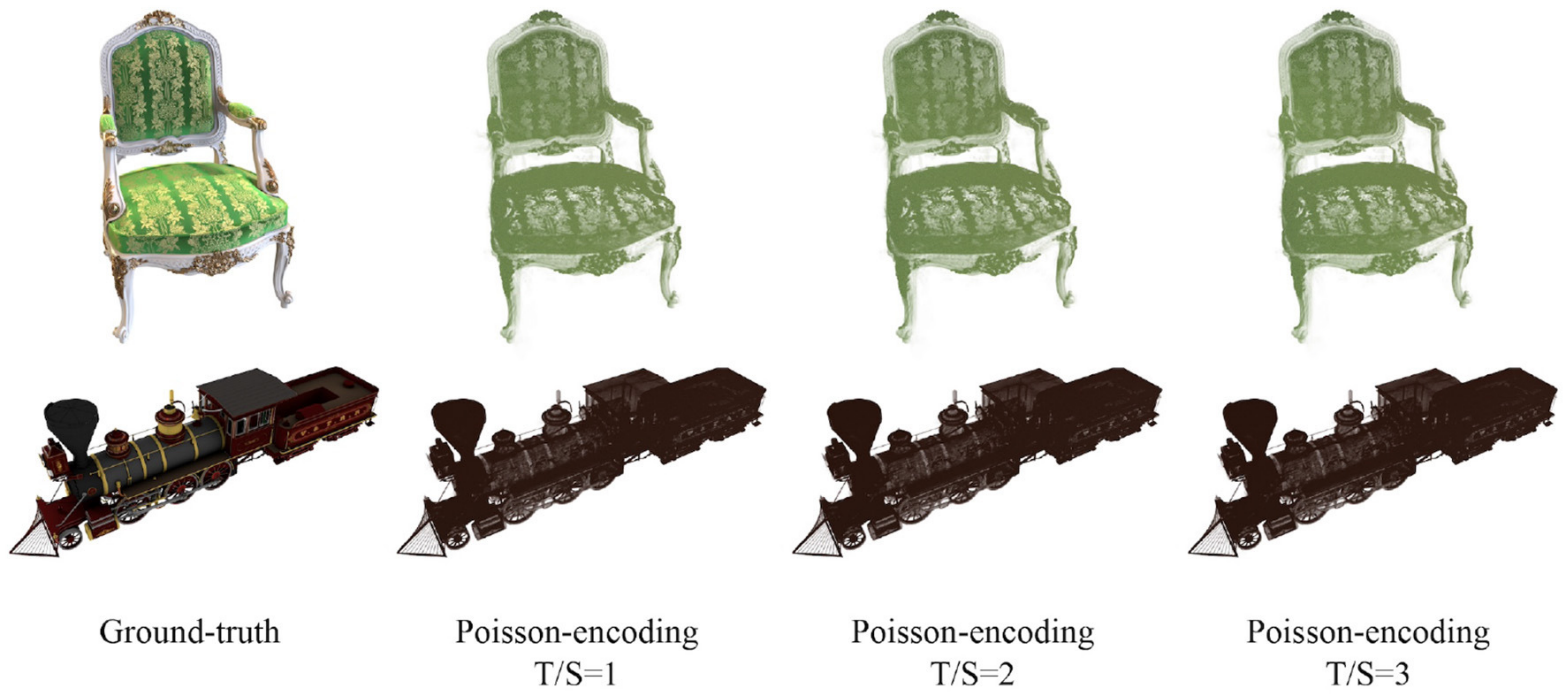
Qualitative results of SpiNeRF-D with Poisson-encoding and different time-steps on *Chair* of Sythetic-NeRF **(top)** and *Steamtrain* of Sythetic-NSVF **(bottom)**. The sampling density is 1.

As described in Section 4.1, we propose two naive versions of SpiNeRF that employ conventional data encoding schemes: Poisson-encoding and direct encoding. As shown in Table 1, Poisson-encoding significantly degrades feature information, yielding a maximum PSNR of only 24.83 dB across different settings and datasets, achieving far-from-acceptable synthesis quality. The corresponding qualitative results of this ineffective encoding are shown in Figure 4. The primary limitation of the Poisson-encoding method is that it leads the NeRF model to capture only dominant color information while discarding much of the finer color detail, ultimately leading to rendering failures. Notably, increasing the temporal length does not alleviate this inherent deficiency. To rule out the possibility that this flaw stems from a low encoding rate, we also calculated the encoding rate. For example, with T/S=3 and D=1, the Poisson encoding rates for Synthetic-NeRF and Synthetic-NSVF are 0.518 and 0.520, respectively both within a reasonable range. A reasonable explanation for this drawback is that Poisson-encoding directly converts full-precision volumetric parameters into binary spikes, bypassing the spiking MLP encoding layer responsible for transforming continuous values into binary spikes. Therefore, a significant part of volumetric information is lost, leading to failure in RGB rendering.

**Table 1:** Comparisons with different data encodings under different time-steps and sampling density settings.

**Method**	**Setting**	**Energy level**	**Synthetic-NeRF**			**Synthetic-NSVF**		
			**PSNR**↑	**SSIM**↑	**Energy (mJ)**↓	**PSNR**↑	**SSIM**↑	**Energy (mJ)**↓
ANN quantization	D=1	1	31.24	0.946	167.67	34.13	0.968	78.54
	D=2	2	31.43	0.949	290.45	34.50	0.971	135.98
	D=3	3	31.50	0.949	506.91	34.53	0.971	239.64
Poisson encoding	T/S=1, D=1	1	22.03	0.854	49.61	24.83	0.893	29.64
	T/S=2, D=1	2	21.98	0.855	91.97	24.83	0.893	55.58
	T/S=3, D=1	3	21.93	0.855	119.04	24.83	0.893	73.04
	T/S=1, D=2	2	22.04	0.857	82.32	24.85	0.894	50.22
	T/S=1, D=3	3	22.03	0.859	136.69	24.85	0.894	88.01
Direct encoding	T/S=1, D=1	1	31.22	0.947	113.03	34.17	0.969	**53.73**
	T/S=2, D=1	2	31.51	0.951	212.20	34.49	0.971	104.05
	T/S=3, D=1	3	31.55	0.951	436.32	34.56	0.971	217.86
	T/S=1, D=2	2	31.40	0.949	192.81	34.45	0.970	**94.58**
	T/S=1, D=3	3	31.46	0.950	337.98	34.56	0.971	168.10
Time-ray alignment (TRA)	T/S=1, D=1	1	**31.34**	**0.949**	**110.80**	**34.33**	**0.970**	56.69
	T/S=1, D=2	2	**31.59**	**0.951**	**185.78**	**34.63**	**0.972**	98.39
	T/S=1, D=3	3	**31.64**	**0.952**	**308.84**	**34.57**	**0.972**	**165.17**

T/S abbreviates Time-step/Sample, representing the number of time-steps of each sampled point. D denotes the normalized sampling density of points along each ray. The bold values indicate better results. Poisson encoding, as a failed study endeavor, is not considered.

Conversely, as listed in Table 1, direct encoding obtains good synthesis performance even with a single time-step, and its PSNR improves as the time-step number increases. Building on this baseline of direct encoding, our proposed TRA encoding shows superior energy efficiency and rendering quality. In the same Table 1, we change TRA's time-step by adjusting the sampling density to compare with direct encoding of different time-steps since it is unfeasible to explicitly set TRA's time-step due to its dynamic temporal length. To ensure a fair comparison across different methods, we introduce the energy level metric, defined as:


(16) $$\begin{array}{l} \text{Energy level}\propto \text{Time-step number of each ray}\\ \;=\text{Time-steps of each sampled point (T/S)}\\ \;\times \text{ sampled point number}\\ \;\propto \text{Time-steps of each sampled point (T/S)}\\ \;\times \text{ Sampling density (D)}. \end{array}$$

Table 1 shows that TRA achieves better rendering quality under the same energy levels. For additional fairness, we also compare TRA with direct encoding under identical sampling densities, and the outcome consistently supports the superiority of TRA.

To further demonstrate the merits of SpiNeRF over efficient ANN data encoding methods such as ANN quantization, we apply the renowned LSQ method (Esser et al., 2020) to quantize DVGO activations to spike-bit, i.e., 1-bit, and compare the results with SpiNeRF-D in Table 1. The results show that SpiNeRF-D outperforms the quantized ANN version on both datasets in terms of synthesis quality and energy efficiency. This demonstrates the superiority of SNNs over ANNs in ultra-low-energy computation scenarios, aligning with findings from previous studies (Deng L. et al., 2020; He et al., 2020). In conclusion, TRA effectively leverages the temporal characteristics of SNNs for 3D rendering, proving a simple yet effective solution.

### 5.2 Comparisons with the ANN counterparts and other NeRFs

#### 5.2.1 Comparisons with ANN-based NeRFs

As shown in Table 2, our SpiNeRF-D with TCP achieves at least 69.82% (56.69 mJ vs. 187.85 mJ, Synthetic-NSVF) and at most 72.95% (581.04 mJ vs. 2147.86 mJ, Tanks&Temples) energy savings on these four datasets over the ANN counterpart. The corresponding PSNR drop is at most 0.78 dB (Synthetic-NSVF). This trade-off between synthesis quality and energy consumption is reasonable, as inference in SpiNeRF-D's spiking MLP relies on addition operations, replacing the more energy-intensive multiplication used in the original DVGO. On the one hand, compared to methods without masking operations, such as NeRF (Mildenhall et al., 2021), Mip-NeRF (Barron et al., 2021), and JaxNeRF (Deng B. et al., 2020), SpiNeRF-D can reach orders of magnitude lower energy consumption. On the other hand, compared to methods that incorporate masking operations, such as NSVF (Liu et al., 2020), DIVeR (Wu et al., 2022), DVGO (Sun et al., 2022), and TensoRF (Chen et al., 2022), SpiNeRF-D still outperforms them in energy efficiency while maintaining comparable synthesis quality. Furthermore, even when compared to KiloNeRF (Reiser et al., 2021), which is optimized for fast rendering but requires days of training, SpiNeRF-D achieves better performance with only minutes of training. Moreover, SpiNeRF-T reduces energy consumption by up to 67.75% (149.98 mJ vs. 465.09 mJ, Synthetic-NSVF) across various datasets. Except for the Tanks&Temples dataset, SpiNeRF-T consistently outperforms DVGO in both PSNR and energy cost. Notably, SpiNeRF-T has only two FC layers, as in TensoRF, one for encoding data with full precision and the other for binary computation using spiking neurons. As a result, only half of the computational workload is handled through addition-based operations, which explains why SpiNeRF-T achieves less energy reduction than SpiNeRF-D. Overall, these results demonstrate the effectiveness of SpiNeRF in improving energy efficiency across different NeRF frameworks.

**Table 2:** Comparisons with the ANN counterpart and other NeRF-based methods.

**Dataset**	**Synthetic-NeRF**			**Synthetic-NSVF**			**BlendedMVS**			**Tanks&temples**		
**Metric**	**PSNR**↑	**SSIM**↑	**Energy**↓**(mJ)**	**PSNR**↑	**SSIM**↑	**Energy**↓**(mJ)**	**PSNR**↑	**SSIM**↑	**Energy**↓**(mJ)**	**PSNR**↑	**SSIM**↑	**Energy**↓**(mJ)**
NeRF (Mildenhall et al., 2021)	31.01	0.947	4.5e5	30.81	0.952	4.5e5	24.15	0.828	3.1e5	25.78	0.864	1.4e6
Mip-NeRF (Barron et al., 2021)	33.09	0.961	4.5e5	-	-	-	-	-	-	-	-	-
JaxNeRF (Deng B. et al., 2020)	31.65	0.952	4.5e5	-	-	-	-	-	-	27.94	0.904	1.4e6
NSVF; Liu et al. (2020)	31.74	0.953	16427	35.13	0.979	8864	26.90	0.898	15149	28.40	0.900	101443
DIVeR (Wu et al., 2022)	32.32	0.960	343.96	-	-	-	27.25	0.910	548.65	28.18	0.912	1930.67
KiloNeRF (Reiser et al., 2021)	31.00	0.95	185.12	33.37	0.97	99.89	27.39	0.92	170.71	28.41	0.91	723.79
DVGO* (Sun et al., 2022)	31.98	0.957	374.72	35.12	0.976	187.85	**28.15**	**0.922**	320.66	28.42	0.912	2147.86
TensoRF* (Chen et al., 2022)	**33.14**	**0.963**	641.17	**36.74**	**0.982**	465.09	-	-	-	**28.50**	**0.920**	2790.03
Spiking-NeRF (Li et al., 2025)	30.41	-	3.7e4^†^	-	-	-	-	-	-	-	-	-
SpiNeRF-D w/ TP	31.34	0.949	111.59	34.34	0.970	57.57	27.80	0.912	97.38	28.00	0.892	**483.48**
SpiNeRF-D w/ TCP	31.34	0.949	**110.80**	34.34	0.970	**56.69**	27.80	0.912	**96.37**	28.09	0.896	581.04
SpiNeRF-T w/ TCP	32.45	0.956	240.81	35.76	0.978	149.98	-	-	-	28.09	0.904	1165.90

*Denotes an ANN counterpart implemented by the official codes. ^†^Denotes a better hardware technology. They use 3.2 pJ/MAC and 0.1 pJ/AC, while we use 4.6 pJ/MAC and 0.9 pJ/AC. Bold values indicate better results.

**Figure 5:**
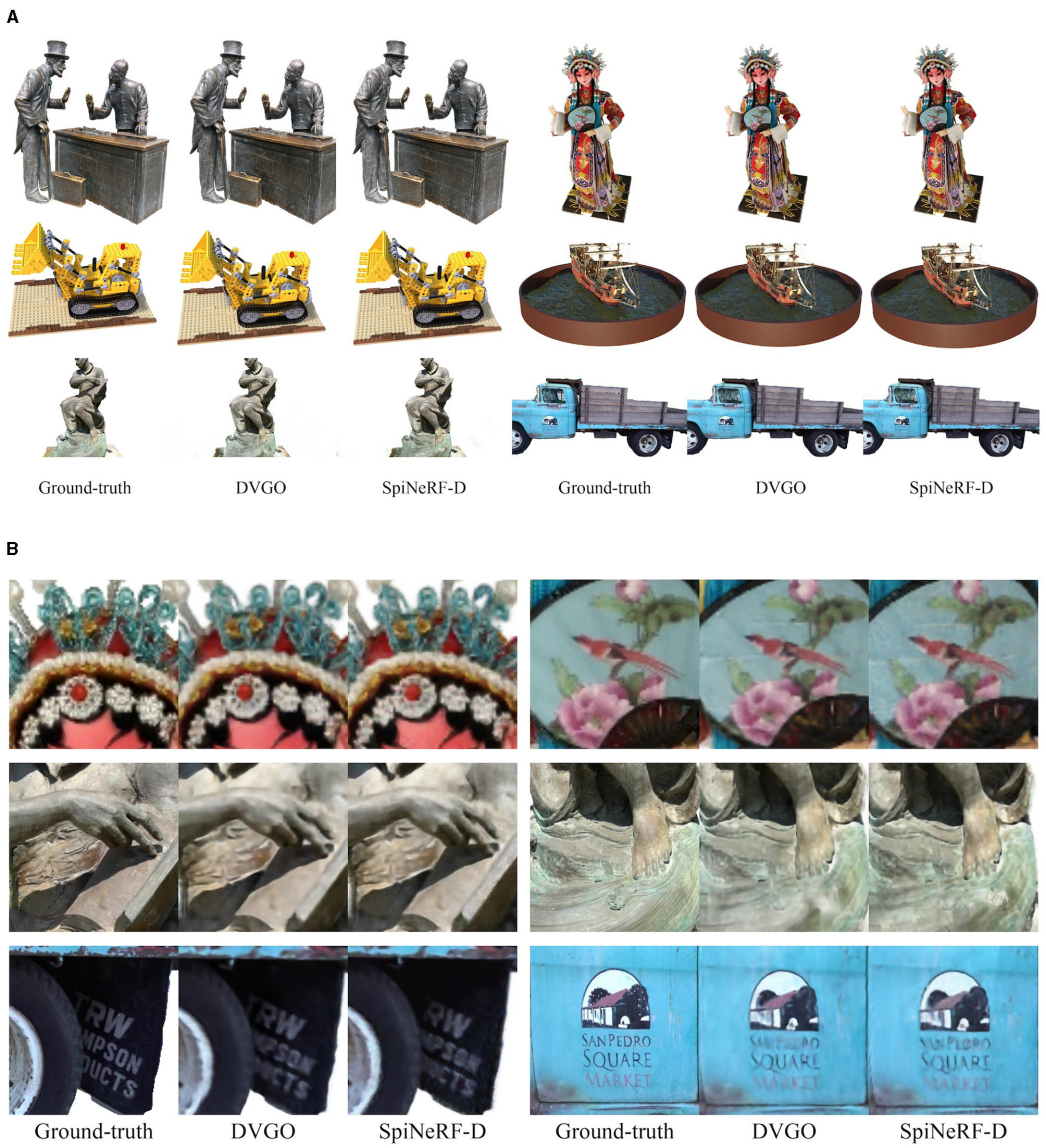
Qualitative results of SpiNeRF-D and the ANN counterpart. **(A)** Qualitative results of six different scenes from synthetic and real datasets. Each row displays two scenes from the same dataset. BlendedMVS, Synthetic-NeRF, and Tanks&Temples are placed from top to bottom by order. **(B)** Zoomed-in images of six challenging parts. Qualitative comparisons on the different challenging parts. Top: On *Character* from BlendedMVS, where the color changes densely and intensely. Middle: On *Ignatius* from Tanks&Temples, where the textures are distinct and dense. Bottom: On *Truck* from Tanks&Temples, where detailed information is explicitly displayed.

Figure 5, which serves as visual aids, demonstrates the effectiveness of our proposed SpiNeRF. Overall, our methods produce comparable rendering quality to their ANN counterparts, as shown in Figure 5A. Figure 5B further compares SpiNeRF-D with its ANN counterpart in six different challenging parts. SpiNeRF-D exhibits similar issues to its ANN counterpart regarding texture distortion and blurring effects.

#### 5.2.2 Comparisons with the SNN-based NeRF

Spiking-NeRF (Li et al., 2025) follows the ANN2SNN conversion strategy and uses direct encoding, to convert an ANN-based NeRF into an SNN version. The ANN2SNN conversion (Diehl et al., 2016; Rueckauer et al., 2017) is built upon the logic that a real-valued activation of ANN can be equivalently converted to a corresponding firing rate of SNN. Therefore, an *x*-bit activation will cost 2^*x*^ time-steps to realize lossless value conversion. One of the inherent limitations of Spiking-NeRF is excessive time-step requirements. As shown in Table 3, Spiking-NeRF requires 256 time-steps per sampled point to achieve comparable PSNR. Another inherent limitation is an inability to leverage SNN's sequential processing capability, as Spiking-NeRF only uses the conventional direct encoding strategy.

**Table 3:** Comparisons with Spiking-NeRF on each scene.

**Scene**	**Spiking-NeRF (Li et al.**, **2025****) (ANN2SNN conversion)**			**Our SpiNeRF (SNN direct training)**		
	**T/S**	**PSNR**↑	**Energy**^†^ **(mJ)** ↓	**T/S**	**PSNR**↑	**Energy (mJ)**↓
Mic	256	32.37	3.56e4	1	**32.47**	**26.48**
Lego	256	30.98	4.41e4	1	**33.82**	**93.20**
Ship	256	**28.77**	3.42e4	1	28.63	**283.45**
Chair	256	32.84	4.10e4	1	**33.36**	**53.67**
Drums	256	**25.23**	3.04e4	1	25.21	**66.99**
Ficus	256	28.91	2.67e4	1	**32.04**	**47.24**
Hotdog	256	35.83	4.83e4	1	**36.00**	**148.49**
Materials	256	28.36	3.64e4	1	**29.15**	**166.88**

T/S abbreviates Time-step/sample, representing the time-step number of each sampled point. ^†^Denotes a better hardware technology. They use 3.2 pJ/MAC and 0.1 pJ/AC, while we use 4.6 pJ/MAC and 0.9 pJ/AC. Bold values indicate better results.

In contrast, our SpiNeRF adopts a direct-training strategy and leverages TRA, achieving an extremely low number of time-steps per sampled point and generally better rendering quality. As shown in Table 3, SpiNeRF's energy consumption remains orders of magnitude lower than that of Spiking-NeRF, despite employing less advanced hardware technology. Another factor contributing to this significant energy efficiency advantage is the use of a hybrid volumetric representation, as described in Section 3.2 and Algorithm 1. This approach significantly reduces the number of sampled points per rendering ray, thereby minimizing the number of spiking MLP queries needed per view.

Additionally, as shown by comparing Tables 2, 3, Spiking-NeRF also fails to surpass certain ANN-based NeRF works in terms of energy efficiency. This is also due to the excessively large number of time-steps discussed above.

### 5.3 Advantages of temporal condensing on hardware architecture

To demonstrate the advantages of the proposed temporal condensing on hardware architecture, as described in Section 4.3, we evaluate SpiNeRF-D with TCP and TP using SpikeSim on the SpikeFlow architecture. As listed in Table 4, TCP consistently outperforms TP in both inference latency and energy overhead across the two datasets. Specifically, TCP demonstrates an order-of-magnitude advantage over TP in both inference speed and energy consumption. These results indicate that TCP achieves efficient inference through a simple mechanism. Comparing results on SpikeSim (65 nm technology, Table 4) with those on 45 nm technology general hardware (Table 2) further demonstrates that the proposed SpiNeRF-D can substantially benefit from its neuromorphic computing nature on neuromorphic hardware, outperforming ANN baselines in energy efficiency. Additionally, temporal condensing will not critically compromise the rendering quality as shown in Table 2. In the SpikeSim evaluation, the temporal condensing operation is done off-chip. This allows on-chip computation to fully benefit from dense data, thereby explaining the significant performance gap between TCP and TP. Note that owing to the pipeline mechanism, the latency of such off-chip operation can be easily covered.

**Table 4:** Comparisons between TCP and TP on SpikeSim using SpiNeRF-D.

**Dataset**	**Synthetic-NeRF**		**Synthetic-NSVF**		**BlendedMVS**		**Tanks&temples**	
**SpiNeRF-D**	**w/ TCP**	**w/ TP**	**w/ TCP**	**w/ TP**	**w/ TCP**	**w/ TP**	**w/ TCP**	**w/ TP**
Latency(s)↓	**26.12**	222.22	**13.37**	164.61	**22.70**	243.93	**138.98**	980.28
Energy^+^(mJ)↓	**65.78**	559.45	**33.68**	414.37	**57.16**	614.13	**350.03**	2468.16

^+^Denotes the energy result particularly produced by SpikeSim. Bold values indicate better results.

**Figure 6:**
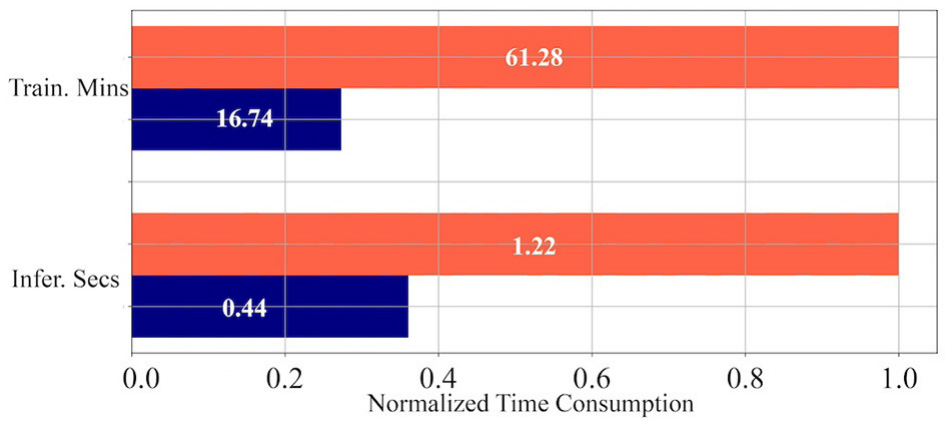
Normalized time consumption of training and inference. Values in the figure represent the average training minutes or inference seconds across each scene. The orange bar is the time consumption of TP, while the blue one is that of TCP.

To evaluate the computational overhead and merits introduced by temporal condensing on general-purpose computing platforms, we showcase the training and inference times on Synthetic-NeRF using a single A100 GPU. As shown in Figure 6, the PyTorch (Imambi et al., 2021) implementation of temporal condensing, even without optimized CUDA kernels, can still achieve significant time reduction for both training and inference on GPU. On average, training time is reduced by 72.68% (16.74 min vs. 61.28 min) and inference time is reduced by 63.93% (0.44 s vs. 1.22 s).

In conclusion, the proposed temporal condensing enhances SpiNeRF's computational efficiency on both neuromorphic and general-purpose hardware architectures.

### 5.4 Discussion of the alignment direction

As described in Section 4.4, we propose the temporal flip to empirically determine the alignment direction, as the querying direction of the spiking MLP along the pixel-rendering ray can affect the inference outcome. Table 5 presents experimental results of SpiNeRF-D with and without temporal flip, i.e., with consistent versus reversed directions. Aligning the temporal dimension with the pixel-rendering ray yields better synthesis performance and energy efficiency across both datasets. This observation is further supported by results shown in Table 6 for the TP-based SpiNeRF-D. Although the temporal flip slightly improves the synthesis quality on Tanks&Temples in the TP-based case, it also causes a significant increase in energy consumption. In most cases, the temporal flip negatively impacts both synthesis quality and energy cost. Therefore, maintaining a consistent alignment direction is important for SpiNeRF.

**Table 5:** Comparisons with temporal flip on TCP-based SpiNeRF-D.

**Dataset**	**Synthetic-NeRF**		**Synthetic-NSVF**		**BlendedMVS**		**Tanks&Temples**	
**SpiNeRF-D**	**w/o TF**	**w/ TF**	**w/o TF**	**w/ TF**	**w/o TF**	**w/ TF**	**w/o TF**	**w/ TF**
PSNR↑	**31.34**	31.25	**34.34**	34.15	**27.80**	27.79	**28.09**	28.05
SSIM↑	**0.949**	0.947	**0.970**	0.967	**0.912**	0.910	**0.896**	0.894
Energy (mJ)↓	**110.80**	116.91	**56.69**	61.08	**96.37**	104.75	**581.04**	612.66

TF, temporal flip. Bold values indicate better results.

**Table 6:** Comparisons with temporal flip on TP-based SpiNeRF-D.

**Dataset**	**Synthetic-NeRF**		**Synthetic-NSVF**		**BlendedMVS**		**Tanks&temples**	
**SpiNeRF-D**	**w/o TF**	**w/ TF**	**w/o TF**	**w/ TF**	**w/o TF**	**w/ TF**	**w/o TF**	**w/ TF**
PSNR↑	**31.34**	31.24	**34.34**	34.06	**27.80**	27.79	28.01	**28.06**
SSIM↑	**0.949**	0.947	**0.970**	0.967	**0.912**	0.909	0.892	**0.894**
Energy (mJ)↓	**111.59**	117.43	57.57	**56.75**	**97.38**	105.51	**483.48**	617.57

TF, temporal flip. Bold values indicate better results.

## 6 Conclusion

In this study, we propose SpiNeRF, a framework that leverages directly trained SNNs to achieve energy efficiency RGB 3D scene rendering. SpiNeRF directly inputs volumetric parameters into SNNs and aligns the temporal dimension with pixel-rendering rays in a consistent direction, establishing a novel and effective integration of SNNs with NeRF-based rendering. To solve the challenge of irregular tensor, we introduce TP and TCP, which enhance data compactness and computational efficiency. Finally, we validate SpiNeRF across various 3D datasets and a hardware simulator and extend it to an alternative NeRF-based framework, demonstrating the effectiveness of our proposed methods. Compared to previous efficient SNN data encodings and ANN quantization, our proposed method consistently achieves superior rendering quality while consuming less energy. In comparison to the previous SNN-based NeRF work, SpiNeRF not only delivers improved rendering results but also reduces energy consumption by orders of magnitude. Despite these substantial gains in energy efficiency, spike-based computation still results in a reduction in rendering performance, which remains an open challenge for future work.

## Data Availability

The original contributions presented in the study are included in the article/supplementary material, further inquiries can be directed to the corresponding author.

## Footnotes

^1^Energy comparisons in Table 2, 3, along with the analysis in Section 5.2.2, demonstrate that this issue significantly undermines the energy-saving potential of SNNs.

## References

[B1] Barron J. T. Mildenhall B. Tancik M. Hedman P. Martin-Brualla R. Srinivasan P. P. (2021). “Mip-nerf: A multiscale representation for anti-aliasing neural radiance fields,” in Proceedings of the IEEE/CVF International Conference on Computer Vision (Piscataway, NJ: IEEE), 5855–5864. 10.1109/ICCV48922.2021.00580

[B2] Chen A. Xu Z. Geiger A. Yu J. Su H. (2022). “Tensorf: tensorial radiance fields,” in European Conference on Computer Vision (Cham: Springer), 333–350.

[B3] Davies M. Srinivasa N. Lin T.-H. Chinya G. Cao Y. Choday S. H.. (2018). Loihi: a neuromorphic manycore processor with on-chip learning. IEEE Micro 38, 82–99. 10.1109/MM.2018.112130359

[B4] Deng B. Barron J. T. Srinivasan P. P. (2020). JaxNerf: An Efficient Jax Implementation of Nerf . Available online at: http://github.com/googleresearch/google-research/tree/master/jaxnerf (Accessed 7, 2023)

[B5] Deng K. Liu A. Zhu J.-Y. Ramanan D. (2022). “Depth-supervised nerf: Fewer views and faster training for free,” in Proceedings of the IEEE/CVF Conference on Computer Vision and Pattern Recognition, 12882–12891.

[B6] Deng L. Wu Y. Hu X. Liang L. Ding Y. Li G.. (2020). Rethinking the performance comparison between snns and anns. Neural Netw. 121, 294–307. 10.1016/j.neunet.2019.09.005 31586857

[B7] Deng S. Li Y. Zhang S. Gu S. (2021). “Temporal efficient training of spiking neural network via gradient re-weighting,” in International Conference on Learning Representations (San Diego, CA: OpenReview.net).

[B8] Diehl P. U. Cook M. (2015). Unsupervised learning of digit recognition using spike-timing-dependent plasticity. Front. Computat. Neurosci. 9:99. 10.3389/fncom.2015.00099 26941637 PMC4522567

[B9] Diehl P. U. Zarrella G. Cassidy A. Pedroni B. U. Neftci E. (2016). “Conversion of artificial recurrent neural networks to spiking neural networks for low-power neuromorphic hardware,” in 2016 IEEE International Conference on Rebooting Computing (ICRC) (Arlington, VA: IEEE).

[B10] Esser S. K. McKinstry J. L. Bablani D. Appuswamy R. Modha D. S. (2020). “Learned step size quantization,” in International Conference on Learning Representations (San Diego, CA: OpenReview.net).

[B11] Fang W. Chen Y. Ding J. Yu Z. Masquelier T. Chen D.. (2023a). Spikingjelly: an open-source machine learning infrastructure platform for spike-based intelligence. Sci. Adv. 9:eadi1480. 10.1126/sciadv.adi1480 37801497 PMC10558124

[B12] Fang W. Yu Z. Chen Y. Huang T. Masquelier T. Tian Y. (2021a). “Deep residual learning in spiking neural networks,” in Advances in Neural Information Processing Systems (Cambridge, MA: MIP Press), 34.

[B13] Fang W. Yu Z. Chen Y. Masquelier T. Huang T. Tian Y. (2021b). “Incorporating learnable membrane time constant to enhance learning of spiking neural networks,” in Proceedings of the IEEE/CVF International Conference on Computer Vision, 2661–2671.

[B14] Fang W. Yu Z. Zhou Z. Chen D. Chen Y. Ma Z.. (2023b). Parallel spiking neurons with high efficiency and ability to learn long-term dependencies. Adv. Neural Inform. Proc. Syst. 36, 53674–53687.

[B15] Fridovich-Keil S. Yu A. Tancik M. Chen Q. Recht B. Kanazawa A. (2022). “Plenoxels: Radiance fields without neural networks,” in Proceedings of the IEEE/CVF Conference on Computer Vision and Pattern Recognition (Cambridge, MA: MIP Press), 5501–5510. 10.1109/CVPR52688.2022.00542

[B16] Garbin S. J. Kowalski M. Johnson M. Shotton J. Valentin J. (2021). “Fastnerf: High-fidelity neural rendering at 200fps,” in Proceedings of the IEEE/CVF International Conference on Computer Vision (Montreal, QC: IEEE), 14346–14355.

[B17] Garg I. Chowdhury S. S. Roy K. (2021). “Dct-snn: using dct to distribute spatial information over time for low-latency spiking neural networks,” in Proceedings of the IEEE/CVF International Conference on Computer Vision (Montreal, QC: IEEE), 4671–4680.

[B18] He W. Wu Y. Deng L. Li G. Wang H. Tian Y.. (2020). Comparing snns and rnns on neuromorphic vision datasets: similarities and differences. Neural Netw. 132, 108–120. 10.1016/j.neunet.2020.08.001 32866745

[B19] Hedman P. Srinivasan P. P. Mildenhall B. Barron J. T. Debevec P. (2021). “Baking neural radiance fields for real-time view synthesis,” in Proceedings of the IEEE/CVF International Conference on Computer Vision (Montreal, QC: IEEE), 5875–5884.

[B20] Horowitz M. (2014). “1.1 computing's energy problem (and what we can do about it),” in 2014 IEEE international solid-state circuits conference digest of technical papers (ISSCC) (San Francisco, CA: IEEE), 10–14.

[B21] Imambi S. Prakash K. B. Kanagachidambaresan G. (2021). “Pytorch,” in Programming with TensorFlow: Solution for Edge Computing Applications, 87–104.

[B22] Kajiya J. T. Von Herzen B. P. (1984). Ray tracing volume densities. ACM SIGGRAPH Comp. Graph. 18:165–174. 10.1145/964965.808594

[B23] Knapitsch A. Park J. Zhou Q.-Y. Koltun V. (2017). Tanks and temples: Benchmarking large-scale scene reconstruction. ACM Trans. Graph. 36, 1–13. 10.1145/3072959.3073599

[B24] Kundu S. Datta G. Pedram M. Beerel P. A. (2021a). “Spike-thrift: Towards energy-efficient deep spiking neural networks by limiting spiking activity via attention-guided compression,” in Proceedings of the IEEE/CVF Winter Conference on Applications of Computer Vision (San Francisco, CA: IEEE), 3953–3962.

[B25] Kundu S. Pedram M. Beerel P. A. (2021b). “Hire-snn: Harnessing the inherent robustness of energy-efficient deep spiking neural networks by training with crafted input noise,” in Proceedings of the IEEE/CVF International Conference on Computer Vision (Montreal, QC: IEEE), 5209–5218.

[B26] Lee J.-J. Zhang W. Li P. (2022). “Parallel time batching: Systolic-array acceleration of sparse spiking neural computation,” in 2022 IEEE International Symposium on High-Performance Computer Architecture (HPCA) (Seoul: IEEE), 317–330.

[B27] Li Y. Guo Y. Zhang S. Deng S. Hai Y. Gu S. (2021). “Differentiable spike: Rethinking gradient-descent for training spiking neural networks,” in Advances in Neural Information Processing Systems (Cambridge, MA: MIP Press), 34.

[B28] Li Z. Ma Y. Zhou J. Zhou P. (2025). Spiking-nerf: Spiking neural network for energy-efficient neural rendering. ACM J. Emerg. Technol. Comp. Syst. 20, 1–23. 10.1145/3675808

[B29] Li Z. Yan B. Li H. (2020). “Resipe: Reram-based single-spiking processing-in-memory engine,” in 2020 57th ACM/IEEE Design Automation Conference (DAC) (San Francisco: IEEE), 1–6.

[B30] Liao Z. Zheng Q. Liu Y. Pan G. (2023). Spiking nerf: Representing the real-world geometry by a discontinuous representation. arXiv [preprint] arXiv:2311.09077. 10.1609/aaai.v38i12.29285

[B31] Lindell D. B. Martel J. N. Wetzstein G. (2021). “Autoint: Automatic integration for fast neural volume rendering,” in Proceedings of the IEEE/CVF Conference on Computer Vision and Pattern Recognition (Nashville, TN: IEEE), 14556–14565.

[B32] Liu L. Gu J. Zaw Lin K. Chua T.-S. Theobalt C. (2020). Neural sparse voxel fields. Adv. Neural Inform. Proc. Syst. 33, 15651–15663.

[B33] Liu Y. Peng S. Liu L. Wang Q. Wang P. Theobalt C.. (2022). “Neural rays for occlusion-aware image-based rendering,” in Proceedings of the IEEE/CVF Conference on Computer Vision and Pattern Recognition (Cambridge, MA: MIP Press), 7824–7833.

[B34] Maass W. (1997). Networks of spiking neurons: the third generation of neural network models. Neural Netw. 10, 1659–1671. 10.1016/S0893-6080(97)00011-7

[B35] Mao R. Tang L. Yuan X. Liu Y. Zhou J. (2024). “Stellar: energy-efficient and low-latency snn algorithm and hardware co-design with spatiotemporal computation,” in 2024 IEEE International Symposium on High-Performance Computer Architecture (HPCA) (Edinburgh: IEEE), 172–185.

[B36] Masland R. H. (2012). The neuronal organization of the retina. Neuron 76, 266–280. 10.1016/j.neuron.2012.10.002 23083731 PMC3714606

[B37] Max N. (1995). Optical models for direct volume rendering. IEEE Trans. Visualizat. Comp. Graph. 1, 99–108. 10.1109/2945.468400

[B38] Mildenhall B. Srinivasan P. P. Tancik M. Barron J. T. Ramamoorthi R. Ng R. (2021). Nerf: Representing scenes as neural radiance fields for view synthesis. Commun. ACM 65, 99–106. 10.1145/3503250

[B39] Moitra A. Bhattacharjee A. Kuang R. Krishnan G. Cao Y. Panda P. (2023). SpikeSim: an end-to-end compute-in-memory hardware evaluation tool for benchmarking spiking neural networks. IEEE Trans. Comp.-Aided Design Integrat. Circuits Syst. 42, 3815–3828. 10.1109/TCAD.2023.3274918

[B40] Neftci E. O. Mostafa H. Zenke F. (2019). Surrogate gradient learning in spiking neural networks: Bringing the power of gradient-based optimization to spiking neural networks. IEEE Signal Proc. Magazine 36, 51–63. 10.1109/MSP.2019.2931595

[B41] Reiser C. Peng S. Liao Y. Geiger A. (2021). “KiloNeRF: Speeding up neural radiance fields with thousands of tiny MLPs,” in Proceedings of the IEEE/CVF International Conference on Computer Vision (Piscataway, NJ: IEEE), 14335–14345. 10.1109/ICCV48922.2021.01407

[B42] Roy K. Jaiswal A. Panda P. (2019). Towards spike-based machine intelligence with neuromorphic computing. Nature 575, 607–617. 10.1038/s41586-019-1677-2 31776490

[B43] Rueckauer B. Lungu I.-A. Hu Y. Pfeiffer M. Liu S.-C. (2017). Conversion of continuous-valued deep networks to efficient event-driven networks for image classification. Front. Neurosci. 11:682. 10.3389/fnins.2017.00682 29375284 PMC5770641

[B44] Sun C. Sun M. Chen H.-T. (2022). “Direct voxel grid optimization: Super-fast convergence for radiance fields reconstruction,” in Proceedings of the IEEE/CVF Conference on Computer Vision and Pattern Recognition (New Orleans, LA: IEEE), 5459–5469.

[B45] Tancik M. Srinivasan P. Mildenhall B. Fridovich-Keil S. Raghavan N. Singhal U.. (2020). Fourier features let networks learn high frequency functions in low dimensional domains. Adv. Neural Inform. Proc. Syst. 33, 7537 –7547.

[B46] Wässle H. (2004). Parallel processing in the mammalian retina. Nat. Rev. Neurosci. 5, 747–757. 10.1038/nrn1497 15378035

[B47] Wizadwongsa S. Phongthawee P. Yenphraphai J. Suwajanakorn S. (2021). “Nex: Real-time view synthesis with neural basis expansion,” in Proceedings of the IEEE/CVF Conference on Computer Vision and Pattern Recognition (Nashville, TN: IEEE), 8534–8543.

[B48] Wu L. Lee J. Y. Bhattad A. Wang Y.-X. Forsyth D. (2022). “Diver: Real-time and accurate neural radiance fields with deterministic integration for volume rendering,” in Proceedings of the IEEE/CVF Conference on Computer Vision and Pattern Recognition, 16200–16209.

[B49] Wu Y. Deng L. Li G. Zhu J. Xie Y. Shi L. (2019). Direct training for spiking neural networks: Faster, larger, better. Proc. AAAI Conf. Artif. Intelligen. 33, 1311–1318. 10.1609/aaai.v33i01.33011311

[B50] Yao M. Zhao G. Zhang H. Hu Y. Deng L. Tian Y.. (2023). Attention spiking neural networks. IEEE Trans. Pattern Analy. Mach. Intellig. 45, 9393–9410. 10.1109/TPAMI.2023.3241201 37022261

[B51] Yao Y. Luo Z. Li S. Zhang J. Ren Y. Zhou L.. (2020). “BlendedMVS: A large-scale dataset for generalized multi-view stereo networks,” in Proceedings of the IEEE/CVF Conference on Computer Vision and Pattern Recognition (Seattle, WA: IEEE), 1790–1799.

[B52] Yu A. Li R. Tancik M. Li H. Ng R. Kanazawa A. (2021). “PlenOctrees for real-time rendering of neural radiance fields,” in Proceedings of the IEEE/CVF International Conference on Computer Vision, 5752–5761.

[B53] Zhang Z. Wang H. Han S. Dally W. J. (2020). “Sparch: efficient architecture for sparse matrix multiplication,” in 2020 IEEE International Symposium on High Performance Computer Architecture (HPCA) (IEEE), 261–274.

[B54] Zheng H. Wu Y. Deng L. Hu Y. Li G. (2021). Going deeper with directly-trained larger spiking neural networks. Proc. AAAI Conf. Artif. Intellig. 35, 11062–11070. 10.1609/aaai.v35i12.17320

[B55] Zhou Z. Zhu Y. He C. Wang Y. Yan S. Tian Y.. (2022). Spikformer: When spiking neural network meets transformer. arXiv [preprint] arXiv:2209.15425. 10.48550/arXiv.2209.15425

[B56] Zhu R.-J. Zhao Q. Eshraghian J. K. (2023). SpikeGPT: Generative pre-trained language model with spiking neural networks. arXiv [preprint] arXiv:2302.13939. 10.48550/arXiv.2302.13939

[B57] Zhu Z. Peng J. Li J. Chen L. Yu Q. Luo S. (2022). Spiking graph convolutional networks. arXiv [preprint] arXiv:2205.02767. 10.24963/ijcai.2022/338

