# Supplementary material for: SpiNeRF: direct-trained spiking neural networks for efficient neural radiance field rendering
Source: Front Neurosci. 2025 Jul 23;19:1593580. doi: 10.3389/fnins.2025.1593580 (PMC12326478; doi:10.3389/fnins.2025.1593580)
Supplement: Supplementary file 1 [file Data_Sheet_1.pdf]

Supplementary Material

1 TECHNICAL APPENDIX GUIDELINE

Following is the Technical appendix to the main text, we introduce how this technical appendix is organized in this section.

1. In **Additional implementation details** 2, we introduce every detail of datasets and the SpiNeRF implementation on the DVGO (Sun et al., 2022) and the TensoRF (Chen et al., 2022) framework, where the principles for experimental fairness are strictly adhered to.
2. In **Energy consumption estimation** 3, we illustrate how to estimate the theoretical energy consumption in our experiments.
3. In **Evaluation on SATA** 4, we further showcase and discuss the evaluation on GPU and SATA (Yin et al., 2022).
4. In **Additional experiment results** 5, we reveal more results of our main experiments and ablation study, including the fairness experiments, the ineffective Poisson-encoding results and extensive statistics for the ablation study.
5. In **Extensive experiments** 6, we demonstrate the effectiveness of SpiNeRF on a different 3D task, and apply SpiNeRF to the TensoRF framework for extensive verification.

This appendix is the extension of the main text results. Some content overlaps are remained.

2 ADDITIONAL IMPLEMENTATION DETAILS

All our experiments are conducted on A100 GPUs, and the reconstruction process (both training and inference) of each scene is assigned to single GPU. In **Reproduction results for fairness**, we further demonstrate the fairness of our implementation by experimentation. All experiments use the same default random seed and are not randomness-dependent, so there is no need to do several runs. Following prior arts of NeRF, we run each experiment for one time.

2.1 Dataset introduction

Here, we give a more sufficient introduction of the datasets we use in the paper. We conduct experiments mainly on the four inward-facing datasets, including Synthetic-NeRF (Mildenhall et al., 2021) that contains eight objects synthesized from realistic images, Synthetic-NSVF (Liu et al., 2020) that contains eight objects synthesized by NSVF, BlendedMVS (Yao et al., 2020) with authentic ambient lighting by blending real images, and Tanks&Temples (Knapitsch et al., 2017) which is a real world dataset.

2.2 Implementation on DVGO

As described in the main text, we produce both SpiNeRF-D and DVGO results with the DVGO official codes. In the inward-facing bounded task, including Synthetic-NeRF, Synthetic-NSVF, BlendedMVS, and Tanks&Temples, we keep all the hyper-parameters as same as the original configuration (Sun et al.,

Table S1. Comparisons between our reproductions and the official results.

Dataset	Synthetic-NeRF		Synthetic-NSVF		BlendedMVS		Tanks&Temples	
Metric	PSNR↑	SSIM↑	PSNR↑	SSIM↑	PSNR↑	SSIM↑	PSNR↑	SSIM↑
Official results collected from their papers.								
DVGO	31.95	0.957	35.08	0.975	28.02	0.922	28.41	0.911
TensoRF	33.14	0.963	36.52	0.982	-	-	28.56	0.920
Our reproduced results.								
DVGO*	31.98	0.957	35.12	0.976	28.15	0.922	28.42	0.912
TensoRF*	31.14	0.963	36.74	0.982	-	-	28.50	0.920

* denotes our reproduced results by the official codes.

2022) except for the training iteration in the fine stage raised from 20000 to 40000 because SNNs usually encounter the under-fitting issue that requires more training iterations to resolve (Fang et al., 2021b,a). The comparison between SpiNeRF-D and DVGO is fair since the training iteration of our reproduced DVGO is also set to 40000. In the inward-facing unbounded and the forward-facing task, we also keep all hyper-parameters, the same with the original configurations for both SpiNeRF-D and our reproduced DVGO. Notably, the grid resolution setting has different options in the DVGO paper, but this paper chooses only one of them, which is 160^3.

2.3 Implementation on TensoRF

We also plug in our SpiNeRF method to the official TensoRF codes (Chen et al., 2022). For both SpiNeRF-T and our reproduced TensorRF, we discard the feature embedding to alleviate the encoding layer's computation burden. Except that, all hyper-parameters are the same as the official configurations.

3 ENERGY CONSUMPTION ESTIMATION

We follow the prior arts (Zhou et al., 2022; Yao et al., 2023; Kundu et al., 2021a,b; Horowitz, 2014) to provide the theoretical energy consumption estimation in 45nm technology (Horowitz, 2014), and report the energy consumption of rendering a novel view on average. The specific method is as follows:

First, the energy cost of the spike-based operations in SNNs are defined as:

$$SOPs = Spike\_num \times Flops, \tag{S1}$$

$$\text{Energy}_{\text{SOPs}} = 0.9pJ \times SOPs, \tag{S2}$$

where $Spike\_num$ denotes the spike number of the input spike train, $Flops$ is the float-point operations of the following computation that one spike will trigger, and $SOPs$ is the number of spike-based operations.

Similarly, the energy consumption of the float-point operations in ANNs is:

$$\text{Energy}_{\text{FLOPs}} = 4.6pJ \times FLOPs, \tag{S3}$$

where $FLOPs$ is the number of the float-point operations.

For a specific NeRF-based work, the total energy consumption of a novel view $\text{Energy}_{\text{tot}}$ is obtained through:

$$\text{Energy}_{\text{tot}} = Pts\_num \times (\text{Energy}_{\text{SOPs}} + \text{Energy}_{\text{FLOPs}}), \tag{S4}$$

where Pts_num is the total number of the sampled points.

Table S2. Comparisons between the TCP-based and the TP-based SpiNeRF-D with more metrics.

Dataset	Synthetic-NeRF			Synthetic-NSVF			BlendedMVS			Tanks&Temples		
Method	TCP	TP	DVGO*	TCP	TP	DVGO*	TCP	TP	DVGO*	TCP	TP	DVGO*
PSNR↑	31.34	31.34	31.98	34.33	34.33	35.12	27.80	27.80	28.15	28.09	28.01	28.42
SSIM↑	0.949	0.949	0.957	0.970	0.970	0.976	0.912	0.912	0.922	0.896	0.892	0.912
LPIP_{Vgg}↓	0.068	0.068	0.053	0.039	0.039	0.032	0.103	0.103	0.099	0.172	0.174	0.153
LPIP_{Alex}↓	0.039	0.039	0.034	0.021	0.021	0.018	0.065	0.065	0.070	0.139	0.142	0.143
Flops (G)	19.34	19.47	81.46	9.89	10.01	40.84	16.79	16.95	69.71	102.92	85.44	466.93
Sops (G)	24.26	24.49	-	12.43	12.80	-	21.24	21.57	-	119.57	100.52	-
Energy (mJ)	110.80	111.59	374.72	56.69	57.57	187.85	96.37	97.38	320.66	581.04	483.47	2147.86

* denotes our reproduced results by the official codes.

Table S3. Comparisons of SpiNeRF (sNeRF) on different platforms on Synthetic-NeRF.

GPU	PSNR	Train. Mins	Infer. Secs
ANN Counterpart	31.98	6.02	0.21
sNeRF-D TP	31.34	61.28	1.22
sNeRF-D TCP	31.34	**16.74**	**0.44**
Neuromorphic Hardware	SER	Energy+	Energy-
sNeRF-D TP	100%	559.45	25.55
sNeRF-D TCP	**11.78%**	**65.78**	**8.70**

+ and - denote the energy produced by SpikeSim and SATA (Yin et al., 2022), respectively.

For the mask-free methods (Mildenhall et al., 2021; Barron et al., 2021; Deng et al., 2020), we can easily obtain the Pts_num for the number of sampling is fixed. For the methods with the masking operation (Wu et al., 2022; Sun et al., 2022; Chen et al., 2022) and SpiNeRF, we count the Pts_num during the runtime. Specially for NSVF (Liu et al., 2020) that uses the masking operation but costs days to train on single scene and does not provide pretrained models, we can hardly afford the computation overhead of training and choose to estimate the Pts_num through:

$$Pts\_num = \rho \cdot Pts\_Num, \tag{S5}$$

where Pts_Num is the samples' number before the masking operation, which is fixed, and ρ is the valid sample ratio obtained through our DVGO practice and the statistics from (Hu et al., 2022). For Synthetic-NeRF, Synthetic-NSVF, BlendedMVS, and Tanks&Temples, we set ρ to 4.04%, 2.18%, 5.39%, and 7.70%, respectively. Such an estimation on Pts_num in Eq. S5 will incur numeric errors between the runtime-counted (real) and the estimated energy cost on NSVF (Liu et al., 2020).

Given all the aforementioned aspects, we exclusively test this estimation method on Synthetic-NeRF and report "Runtime-counted(mJ) v.s. Estimated(mJ)", which turns out to be "45832 v.s. 16427". Despite the error rate being 64.16%, both the Runtime-counted and Estimated energy costs remain at the same numeric magnitude. While, the gap between SpiNeRF-D and NSVF in energy consumption spans orders of magnitudes as illustrated in the main text. Therefore, the advantages of SpiNeRF over NSVF in energy consumption should be considered definitive. Note that, we only report the estimated energy cost of NSVF, and the energy costs of other mask-applied works are all runtime-counted.

With the above methods, we can quantitatively compare SpiNeRF with its ANN counterparts in energy consumption to show the energy merits SpiNeRF brings.

4 EVALUATION ON SATA

To further demonstrate the effectiveness of our proposed TCP, we further discuss how TCP can be so beneficial to hardware. **A)** For any hardware accelerator and even for GPUs (Table S3), a regular and condensed data structure commonly brings far more benefits to efficiency even if condensing has extra overhead. Such benefits also apply to neuromorphic hardware, as proposed and proved in (Lee et al., 2022) (published at a tier-1 hardware conference). **B)** Not all neuromorphic hardware is event-driven, i.e., sparsity-aware, taking SpikeSim for example. This hardware is not able to skip zero data and has to strictly follow the predefined data flow. That's why TCP producing a more effective data flow could benefit greatly on SpikeSim (65.78 vs 559.45). Such benefit is quantitatively correlated to the Sample Execution Ratio (SER), which refers to the ratio of samples involved in NeRF rendering. As listed in Table S3, 100% samples are processed in TP where zero data are interspersed within while TCP only requires 11.78% where zero data are condensed and discarded. **C)** Even for event-driven hardware (e.g., SATA) that can skip zero data, "no event" (zero data) still costs energy to maintain the dynamics update and sparsity-aware modules. In this hardware, TCP, i.e. low SER, would not benefit so greatly but still help a lot as listed in Table S3 (8.70 vs 25.55).

Additionally, in the SATA evaluation, we do not change the default configuration of the official implementation. We simply mapping the SpiNeRF-D to SATA and report the average energy consumption of the ray rendering process.

5 ADDITIONAL EXPERIMENT RESULTS

5.1 Reproduction results for fairness

Table S1 lists the results of our reproduced DVGO and TensoRF along with the official results to confirm the fairness and correctness of our experiments concerning the ANN counterparts. All experiments use the same default random seed, so there is no need to do several runs, following prior arts of NeRF.

5.2 More results under different metrics

As listed in Table S2, we report more experimental results of SpiNeRF-D and DVGO under different metrics. Most rendering energy of SpiNeRF-D is consumed in the encoding layer where float-operation is performed. And, the synthesis quality of SpiNeRF-D falls behind the ANN counterpart by a small margin under different quality metrics. Notably, the proposed TCP and TP show the same-level synthesis quality and energy consumption. That is why a more accurate estimation on SpikeSim is needed to demonstrate the advantage of TCP.

5.3 The ineffective Poisson-encoding

To avoid the over-length of the main text, we move the results of the ineffective Poisson-encoding to this section. As listed in Table S4, the Poisson-encoding scheme shows at most 24.83 PSNR among all the four datasets, and none of these results shows valid 3D rendering quality. Even with the increasing time steps, the synthesis performance will not climb up as the direct-encoding and the proposed TRA do.

5.4 Comparisons with direct-encoding on specific scenes

In the main text, we only show the averaged PSNR, SSIM, and energy of our SpiNeRF. To demonstrate the overall merits of our proposed TRA and reveal more experimental results, we showcase every PSNR,

Table S4. Results of Poisson-encoding on different datasets with different time steps.

Dataset	Synthetic-NeRF		Synthetic-NSVF		BlendedMVS		TanksTemples	
Metric	PSNR↑	SSIM↑	PSNR↑	SSIM↑	PSNR↑	SSIM↑	PSNR↑	SSIM↑
Poisson-encoding T=1	22.03	0.854	24.83	0.893	20.74	0.759	21.53	0.810
Poisson-encoding T=2	21.98	0.855	24.83	0.893	20.74	0.759	21.57	0.814
Poisson-encoding T=4	21.90	0.856	24.83	0.893	20.74	0.759	21.60	0.818

T denotes the time step.

Table S5. Comparisons on Mip-NeRF360.

Dataset	Mip-NeRF360	
Method	SpiNeRF-D	DVGO*
PSNR↑	24.66	25.41
SSIM↑	0.646	0.694
LPIP$_{Vgg}$↓	0.461	0.430
LPIP$_{Alex}$↓	0.364	0.372
Energy (mJ)	831.43	2113.46

* denotes our reproduced results by the official codes.

SSIM, and energy of SpiNeRF-D with TRA and direct-encoding on each scene of Synthetic-NeRF as the extensive results for the ablation study of **"Comparisons with the conventional data encodings"**.

In Table S7 and Table S8, we compare time-ray alignment (TRA) with direct-encoding (DE) with different time steps on each scene of Synthetic-NeRF. In Table S9 and Table S10, we compare time-ray alignment (TRA) with direct-encoding (DE) with different density levels on each scene of Synthetic-NeRF. These four tables extend the statistics of the original two tables of the main text and still prove our proposed TRA can consistently outperform the conventional direct-encoding scheme.

6 EXTENSIVE EXPERIMENTS

6.1 Extension to another 3D task

As shown in Table S5, we also conduct experiments on SpiNeRF-D on the inward-facing unbounded dataset Mip-NeRF360 (Barron et al., 2022) to demonstrate the effectiveness of SpiNeRF on different kinds of 3D reconstruction. In terms of synthesis quality, SpiNeRF-D works well with a small margin to the ANN counterpart as verified before. As for the energy merits, SpiNeRF-D outperforms DVGO by 60.66% in energy reduction on Mip-NeRF360.

6.2 Extension to TensoRF

In Table S6, we list detailed results of SpiNeRF-T and our reproduced TensoRF. Similar to the case of SpiNeRF-D and DVGO, SpiNeRF-T achieves significant energy reduction with small synthesis performance drop.

Table S6. Extension to the TensoRF framework.

Dataset	Synthetic-NeRF		Synthetic-NSVF		Tanks&Temples	
Method	SpiNeRF-T	TensoRF*	SpiNeRF-T	TensoRF*	SpiNeRF-T	TensoRF*
PSNR↑	32.45	33.14	35.76	36.74	28.09	28.50
SSIM↑	0.956	0.963	0.978	0.982	0.904	0.920
LPIP_{Vgg}↓	0.031	0.028	0.014	0.011	0.124	0.124
LPIP_{Alex}↓	0.058	0.047	0.031	0.025	0.163	0.141
Flops (G)	42.10	139.39	25.95	101.11	207.01	606.53
Sops (G)	52.42	-	34.03	-	237.39	-
Energy (mJ)	240.81	641.17	149.98	465.09	1165.90	2790.03

* denotes our reproduced results by the official codes.

Table S7. Comparisons between TRA and Direct-encoding (DE) with different time steps on the first four scenes of Synthetic-NeRF.

Scene	Drums			Ficus			Hotdog			Ship		
Metric	PSNR↑	SSIM↑	Energy↓	PSNR↑	SSIM↑	Energy↓	PSNR↑	SSIM↑	Energy↓	PSNR↑	SSIM↑	Energy↓
TRA D=1	**25.21**	**0.922**	66.99	32.04	0.973	**47.24**	**36.01**	**0.973**	**148.49**	**28.63**	**0.867**	**283.45**
TRA D=2	**25.36**	**0.925**	**120.21**	**32.29**	0.975	**84.80**	**36.19**	**0.975**	**234.84**	28.81	**0.871**	**468.67**
TRA D=4	**25.39**	**0.926**	**218.28**	**32.39**	**0.976**	**151.11**	**36.23**	0.975	**382.15**	**28.86**	**0.873**	**773.86**
DE T=1	25.19	0.921	**66.54**	**32.07**	0.973	47.94	35.68	0.970	157.66	28.57	0.865	291.80
DE T=2	25.28	0.923	128.60	32.26	0.975	93.86	36.03	0.974	278.16	28.81	0.869	575.61
DE T=4	25.31	0.924	272.50	32.24	0.974	192.86	36.21	0.975	561.47	28.77	0.870	1190.38

Table S8. Comparisons between TRA and Direct-encoding (DE) with different time steps on the last four scenes of Synthetic-NeRF.

Scene	Lego			Materials			Mic			Chair		
Metric	PSNR↑	SSIM↑	Energy↓	PSNR↑	SSIM↑	Energy↓	PSNR↑	SSIM↑	Energy↓	PSNR↑	SSIM↑	Energy↓
TRA D=1	**33.82**	**0.968**	**93.20**	**29.15**	**0.942**	**166.88**	32.47	0.977	**26.48**	**33.36**	**0.969**	53.67
TRA D=2	**34.16**	**0.971**	**150.45**	29.27	0.944	**305.32**	**32.85**	0.979	**43.04**	**33.69**	0.972	**81.21**
TRA D=4	**34.22**	**0.971**	**253.52**	29.21	0.942	**481.77**	**32.98**	0.980	**74.48**	**33.81**	**0.973**	**135.56**
DE T=1	33.53	0.965	96.35	29.05	0.938	167.06	**32.49**	0.977	26.64	33.15	0.968	**50.28**
DE T=2	34.06	0.970	172.93	**29.28**	0.944	314.55	32.76	0.979	48.69	33.60	0.972	85.13
DE T=4	34.07	0.970	351.84	**29.30**	**0.944**	645.10	32.84	0.980	100.08	33.67	0.972	176.29

Table S9. Comparisons between TRA and Direct-encoding (DE) with density levels on the first four scenes of Synthetic-NeRF.

Scene	Drums			Ficus			Hotdog			Ship		
Metric	PSNR↑	SSIM↑	Energy↓	PSNR↑	SSIM↑	Energy↓	PSNR↑	SSIM↑	Energy↓	PSNR↑	SSIM↑	Energy↓
TRA D=1	**25.21**	**0.922**	66.99	32.04	0.973	**47.24**	**36.01**	**0.973**	**148.49**	**28.63**	**0.867**	**283.45**
TRA D=2	**25.36**	**0.925**	120.21	**32.29**	**0.975**	84.80	**36.19**	**0.975**	**234.84**	**28.81**	**0.871**	**468.67**
TRA D=4	**25.39**	**0.926**	218.28	**32.39**	**0.976**	151.11	**36.23**	**0.975**	**382.15**	**28.86**	**0.873**	**773.86**
DE D=1	25.19	0.921	**66.54**	**32.07**	0.973	47.94	35.68	0.970	157.66	28.57	0.865	291.80
DE D=2	25.27	0.923	**119.08**	32.21	0.974	**83.10**	35.78	0.975	259.51	28.79	0.869	491.15
DE D=4	25.31	0.923	**211.05**	32.22	0.974	**149.26**	35.88	0.972	451.66	28.80	0.870	840.95

Table S10. Comparisons between TRA and Direct-encoding (DE) with density levels on the last four scenes of Synthetic-NeRF.

Scene	Lego			Materials			Mic			Chair		
Metric	PSNR↑	SSIM↑	Energy↓	PSNR↑	SSIM↑	Energy↓	PSNR↑	SSIM↑	Energy↓	PSNR↑	SSIM↑	Energy↓
TRA D=1	**33.82**	**0.968**	**93.20**	**29.15**	**0.942**	**166.88**	32.47	0.977	**26.48**	**33.36**	**0.969**	53.67
TRA D=2	**34.16**	**0.971**	**150.45**	**29.27**	**0.944**	**305.32**	**32.85**	0.979	**43.04**	**33.69**	**0.972**	81.21
TRA D=4	**34.22**	**0.971**	**253.52**	**29.21**	**0.942**	**481.77**	**32.98**	**0.980**	**74.48**	**33.81**	**0.973**	**135.56**
DE D=1	33.53	0.965	96.35	29.05	0.938	167.06	**32.49**	0.977	26.64	33.15	0.968	**50.28**
DE D=2	33.83	0.968	156.82	29.04	0.939	306.97	32.74	0.979	46.13	33.52	0.971	**79.74**
DE D=4	33.93	0.969	272.69	29.08	0.939	557.81	32.78	0.979	81.42	33.64	0.972	138.12

REFERENCES

Barron, J. T., Mildenhall, B., Tancik, M., Hedman, P., Martin-Brualla, R., and Srinivasan, P. P. (2021). Mip-nerf: A multiscale representation for anti-aliasing neural radiance fields. In *Proceedings of the IEEE/CVF International Conference on Computer Vision*. 5855–5864

Barron, J. T., Mildenhall, B., Verbin, D., Srinivasan, P. P., and Hedman, P. (2022). Mip-nerf 360: Unbounded anti-aliased neural radiance fields. In *Proceedings of the IEEE/CVF Conference on Computer Vision and Pattern Recognition*. 5470–5479

Chen, A., Xu, Z., Geiger, A., Yu, J., and Su, H. (2022). Tensorf: Tensorial radiance fields. In *European Conference on Computer Vision* (Springer), 333–350

Deng, B., Barron, J. T., and Srinivasan, P. P. (2020). Jaxnerf: an efficient jax implementation of nerf. *URL http://github. com/googleresearch/google-research/tree/master/jaxnerf*

Fang, W., Yu, Z., Chen, Y., Huang, T., Masquelier, T., and Tian, Y. (2021a). Deep residual learning in spiking neural networks. *Advances in Neural Information Processing Systems* 34

Fang, W., Yu, Z., Chen, Y., Masquelier, T., Huang, T., and Tian, Y. (2021b). Incorporating learnable membrane time constant to enhance learning of spiking neural networks. In *Proceedings of the IEEE/CVF International Conference on Computer Vision*. 2661–2671

Horowitz, M. (2014). 1.1 computing's energy problem (and what we can do about it). In *2014 IEEE international solid-state circuits conference digest of technical papers (ISSCC)* (IEEE), 10–14

Hu, T., Liu, S., Chen, Y., Shen, T., and Jia, J. (2022). Efficientnerf efficient neural radiance fields. In *Proceedings of the IEEE/CVF Conference on Computer Vision and Pattern Recognition*. 12902–12911

Knapitsch, A., Park, J., Zhou, Q.-Y., and Koltun, V. (2017). Tanks and temples: Benchmarking large-scale scene reconstruction. *ACM Transactions on Graphics (ToG)* 36, 1–13

Kundu, S., Datta, G., Pedram, M., and Beerel, P. A. (2021a). Spike-thrift: Towards energy-efficient deep spiking neural networks by limiting spiking activity via attention-guided compression. In *Proceedings of the IEEE/CVF Winter Conference on Applications of Computer Vision*. 3953–3962

Kundu, S., Pedram, M., and Beerel, P. A. (2021b). Hire-snn: Harnessing the inherent robustness of energy-efficient deep spiking neural networks by training with crafted input noise. In *Proceedings of the IEEE/CVF International Conference on Computer Vision*. 5209–5218

Lee, J.-J., Zhang, W., and Li, P. (2022). Parallel time batching: Systolic-array acceleration of sparse spiking neural computation. In *2022 IEEE International Symposium on High-Performance Computer Architecture (HPCA)* (IEEE), 317–330

Liu, L., Gu, J., Zaw Lin, K., Chua, T.-S., and Theobalt, C. (2020). Neural sparse voxel fields. *Advances in Neural Information Processing Systems* 33, 15651–15663

Mildenhall, B., Srinivasan, P. P., Tancik, M., Barron, J. T., Ramamoorthi, R., and Ng, R. (2021). Nerf: Representing scenes as neural radiance fields for view synthesis. *Communications of the ACM* 65, 99–106

Sun, C., Sun, M., and Chen, H.-T. (2022). Direct voxel grid optimization: Super-fast convergence for radiance fields reconstruction. In *Proceedings of the IEEE/CVF Conference on Computer Vision and Pattern Recognition*. 5459–5469

Wu, L., Lee, J. Y., Bhattad, A., Wang, Y.-X., and Forsyth, D. (2022). Diver: Real-time and accurate neural radiance fields with deterministic integration for volume rendering. In *Proceedings of the IEEE/CVF Conference on Computer Vision and Pattern Recognition*. 16200–16209

Yao, M., Zhao, G., Zhang, H., Hu, Y., Deng, L., Tian, Y., et al. (2023). Attention spiking neural networks. *IEEE transactions on pattern analysis and machine intelligence*

Yao, Y., Luo, Z., Li, S., Zhang, J., Ren, Y., Zhou, L., et al. (2020). Blendedmvs: A large-scale dataset for generalized multi-view stereo networks. In *Proceedings of the IEEE/CVF conference on computer vision and pattern recognition*. 1790–1799

Yin, R., Moitra, A., Bhattacharjee, A., Kim, Y., and Panda, P. (2022). Sata: Sparsity-aware training accelerator for spiking neural networks. *IEEE Transactions on Computer-Aided Design of Integrated Circuits and Systems* 42, 1926–1938

Zhou, Z., Zhu, Y., He, C., Wang, Y., Yan, S., Tian, Y., et al. (2022). Spikformer: When spiking neural network meets transformer. *arXiv preprint arXiv:2209.15425*
